# Role of X chromosome and dosage-compensation mechanisms in complex trait genetics

**DOI:** 10.1016/j.ajhg.2025.04.004

**Published:** 2025-05-12

**Authors:** Yu Fu, Aino Kenttämies, Sanni Ruotsalainen, Matti Pirinen, Taru Tukiainen

**Affiliations:** 1 Institute for Molecular Medicine Finland (FIMM), Helsinki Institute of Life Science (HiLIFE), University of Helsinki, 00014 Helsinki, Finland; 2 Department of Public Health, University of Helsinki, 00014 Helsinki, Finland; 3 Department of Mathematics and Statistics, University of Helsinki, 00014 Helsinki, Finland

**Keywords:** X chromosome, GWAS, complex traits, dosage compensation, X chromosome inactivation, XCI, X upregulation

## Abstract

The X chromosome (chrX) is often excluded from genome-wide association studies due to its unique biology complicating the analysis and interpretation of genetic data. Consequently, the influence of chrX on human complex traits remains debated. Here, we systematically assessed the relevance of chrX and the effect of its biology on complex traits by analyzing 48 quantitative traits in 343,695 individuals in UK Biobank with replication in 412,181 individuals from FinnGen. We show that, in the general population, chrX contributes to complex trait heritability at a rate of 3% of the autosomal heritability, consistent with the amount of genetic variation observed in chrX. We find that a pronounced male bias in chrX heritability supports the presence of near-complete dosage compensation between sexes through X chromosome inactivation (XCI). However, we also find subtle yet plausible evidence of escape from XCI contributing to human height. Assuming full XCI, the observed chrX contribution to complex trait heritability in both sexes is greater than expected given the presence of only a single active copy of chrX, mirroring potential dosage compensation between chrX and the autosomes. We find this enhanced contribution attributable to systematically larger active allele effects from chrX compared to autosomes in both sexes, independent of allele frequency and variant deleteriousness. Together, these findings support a model in which the two dosage-compensation mechanisms work in concert to balance the influence of chrX across the population while preserving sex-specific differences at a manageable level. Overall, our study advocates for more comprehensive locus discovery efforts in chrX.

## Graphical abstract

**Figure:**
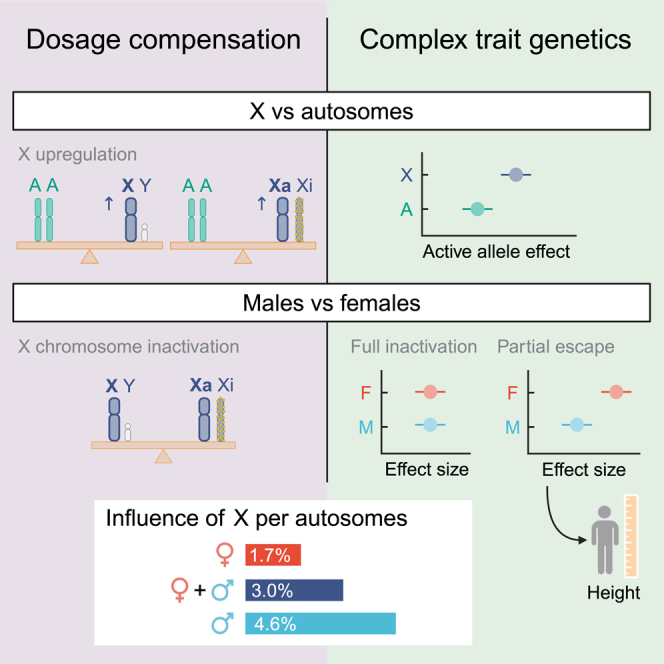

## Teaser

We show that the X chromosome, understudied in genome-wide association studies, contributes to complex traits in a manner shaped by dosage-compensation mechanisms. We find near-full X inactivation balancing the dosage between males and females, with evidence for escape from X inactivation influencing height, and suggest partial dosage compensation between the X chromosome and autosomes.

## Introduction

Genome-wide association studies (GWASs) have discovered numerous autosomal variants associated with complex traits and diseases. However, the discovery of phenotype-associated X chromosome (chrX) loci is significantly lagging behind autosomes, despite chrX constituting ∼5% of the human genome and harboring at least 800 protein-coding genes. Indeed, it was estimated that only 25% of the published GWASs reported a chrX analysis in the NHGRI-EBI GWAS Catalog^1^ in 2021,^2^ thus leaving the contribution of chrX to genetics of complex phenotypes largely unexplored. Nevertheless, genetic studies that have analyzed chrX have showcased its non-negligible role in many complex phenotypes and that novel biological discoveries can be uncovered from this chromosome.^3^^,^^4^^,^^5^^,^^6^^,^^7^^,^^8^^,^^9^

One of the major contributors to the exclusion of chrX has been the analytical and interpretational challenges posed by the unique biology of chrX.^2^^,^^10^ Unlike autosomes that occur in pairs, in most mammals, including humans, chrX is present as two copies in genetic females (XX karyotype) but only as one copy in genetic males (XY karyotype). This leaves the extensive non-pseudoautosomal region (non-PAR) of chrX hemizygous in males. To counter the putative dosage imbalance, chrX-specific regulatory processes act to compensate for the differences in chrX dosage between males and females as well as between chrX and autosomes. Ohno proposed in 1967^11^ that dosage compensation is initiated during embryogenesis and functions through two mechanisms: (1) random X chromosome inactivation (XCI) in each female somatic cell to equalize the active dosage between sexes, leaving chrX functionally hemizygous also in XX cells; and (2) 2-fold upregulation of X-linked gene expression compared to autosomal genes to balance the dosage difference between one active chrX and a pair of active autosomes.

Since first hypothesized in the 1960s by Mary Lyon,^12^ XCI has now been accepted as a fact,^13^ resulting in broadly equal levels of gene expression between sexes.^14^^,^^15^ However, in humans, as many as 25% of chrX genes escape from XCI and continue to be expressed at attenuated level from the inactive chrX.^15^^,^^16^^,^^17^ In contrast to XCI, the evidence supporting X upregulation is more conflicting. Gene-expression evidence generally converges to proposing partial transcriptional upregulation of chrX genes across various organisms including humans^18^^,^^19^^,^^20^^,^^21^^,^^22^^,^^23^ in a manner whereby the expression from a single copy of chrX is greater than that from a single autosome but lower than that from an autosome pair. Whether a similar compensatory process between chrX and autosomes extends to other biological layers, including genetic effects on complex traits, remains unclear.

Large-scale genetic data have the potential to elaborate on the male-to-female and chrX-to-autosome relationships in human complex traits in the light of dosage compensation. Full XCI is expected to manifest as a 2-fold additive genetic variance in males compared to females.^24^ Accordingly, comparisons of chrX SNP heritabilities ($h_{X}^{2}$) between males and females have generally supported the presence of XCI across diverse complex traits.^4^^,^^5^ Escape from XCI, is, theoretically, expected to modify this relationship and lead to a subtle increase in female $h_{X}^{2}$ and female genetic effects. However, contrasting the transcriptome-level evidence of widespread escape,^15^^,^^16^^,^^25^ genetic studies, which have usually assumed complete absence of XCI at escape loci, have provided limited support for the contribution of escape to human complex traits.^4^ Genetic studies that compared chrX and autosomes have found the effects of chrX to be smaller than those of a pair of autosomes in females^26^ but comparable to those of a single autosome in males.^4^ Most GWAS tools nowadays support chrX analyses, facilitating the examination of chrX contributions to human complex traits. However, the inference about dosage-compensation mechanisms is complicated by differing assumptions underlying these tools regarding male-to-female and chrX-to-autosome relationships (see complications and consequences of chrX biology in GWASs and supplemental notes).

In this study, we addressed the complication in understanding chrX GWAS results due to the unique biology of chrX. We leveraged data on genotypes and 48 complex traits from 159,112 males and 184,583 females in the UK Biobank (UKB)^27^ with replication data from 181,871 males and 230,310 females in FinnGen^28^ (release 10). We surveyed the contribution of chrX across the complex traits in the overall population and within each sex through partitioning SNP heritability between autosomes and chrX. We extended our study to understand how the unique biology of chrX is reflected in the phenotypic associations through comparison of sex bias in heritabilities and genetic effects in chrX versus the autosomes. Altogether, we provide insight into the effects of chrX-specific biology on GWASs and the importance of accounting the unique features of chrX in the analysis and interpretation of genetic studies.

## Material and methods

### Complications and consequences of chrX biology in GWASs

The analysis of non-PAR in chrX poses several analytical challenges due to differing copy numbers of chrX between sexes and XCI in females. Here, we first explain the motivation and consequence of the most commonly adopted system in GWAS tools^29^^,^^30^^,^^31^^,^^32^ that codes female genotypes as {0,1,2} and male as {0,2} (other coding systems are explained in supplemental notes) in non-PAR. We then discuss the power bias in sex-specific and sex-combined GWASs introduced by different copy numbers of chrX between sexes. Last, we discuss the expectations of genetic variance, heritability, and effect sizes considering different degrees of escape from XCI. The effect sizes are determined through a linear regression model Y∼Gβ+covariates, where trait Y is regressed on genotype G to estimate the effect size β. We use a to denote the active allele effect.

Random XCI in females results in ∼50% cells with maternal chrX active and ∼50% with paternal chrX active. Thus, assuming full XCI in females, homozygous loci (aa or AA) in a female cell are functionally equal to a hemizygous male cell with the same allele, while heterozygous loci (aA) typically have allele A functionally active in ∼50% cells and allele a in the other ∼50% of the cells.

**Figure 1:**
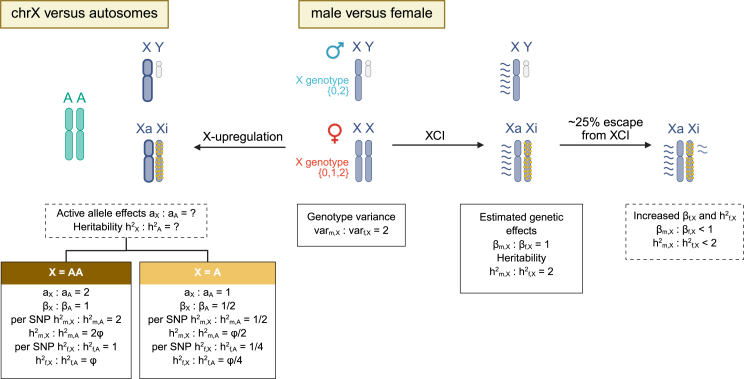
Consequences of dosage compensation in the analysis and interpretation of genetic association data in chrX The assumptions are based on the model in which the chrX genotypes in females are coded as {0,1,2} and in males as {0,2}. XCI, X chromosome inactivation; Xa, active X chromosome; Xi, inactive X chromosome; $\beta_{f,X},\beta_{m,X}$, female and male GWAS effects in chrX, respectively; $h_{f,X}^{2},h_{m,X}^{2}$, chrX heritabilities in females and males, respectively; $a_{X},a_{A}$, active allele effects in chrX and autosomes, respectively; $h_{f,A}^{2},h_{m,A}^{2}$, autosomal heritabilities in females and males, respectively; ϕ, the ratio of number of variants contributing to heritability in chrX versus that in autosomes. X = AA and X = A denote scenarios where the effect of one active chrX is comparable to that of a pair of autosomes and a single autosome, respectively.

The linear model effect sizes in males ($\beta_{m})$ and females ($\beta_{f}$) are equal if we assume full XCI and equal active allele effect sizes (a) between the sexes. When both sexes are analyzed together, we are implicitly assuming that one of the alleles in females is fully inactivated^33^ and the estimated effect size parameter denotes half the effect of an active allele (i.e., $\beta_{m}=\beta_{f}=a_{X}/2$). When the functionally haploid chrX causes similar magnitude of phenotypic effect as a pair of the autosome (X = AA), the estimated effects between variants in chrX and in an autosome are expected to be equal. In contrast, if one active chrX causes similar magnitude of effect as a single autosome (X = A), the estimated effects of variants in chrX are expected to be half of those of autosomal variants. We summarize the relationship between males and females, and chrX and autosomes, for different quantities in Figure 1 and Table S1.

The difference in ploidies between sexes is an inherent nature of chrX. Consequently, assuming full XCI and the same active allele effect in both sexes, the trait variance explained by a genetic locus ($\beta^{2}\text{var}\left(G\right)$) is twice as large in males as in females (Figure 1 and Table S1). Thus, statistical power to detect a non-zero effect, which is an increasing function of the variance explained by the locus, is also larger in males than in females (assuming non-genetic variance of the trait is similar between the sexes). When performing a sex-specific analysis, the number of significant loci in male GWASs is expected to be larger than that in female GWASs in chrX given equal effect sizes, minor allele frequency (MAF), and sample sizes. For sex-combined GWASs, a common approach to carry out such analysis is a fixed-effect meta-analysis of sex-specific GWASs assuming $\beta_{f}=\beta_{m}=\beta$. For a chrX locus, whose effect size differs between the sexes, this analysis has larger power to detect male-biased loci ($\left|\beta_{m}\right|>\;\left|\beta_{f}\right|$) than female-biased loci ($\left|\beta_{f}\right|>\;\left|\beta_{m}\right|$). A symmetric detection of sex-biased effect sizes in chrX under full XCI would require the sample size of males to be half of that of females to compensate for the doubled variance explained in males. We exemplify the power bias between the sexes in Figure S1.

The escape from XCI is predicted to result in an increase in female effect compared to male effect at a locus level and, thus, also result in an increased additive genetic variance in females.^4^ Therefore, at a variant level, we can search for potential escape regions among those regions where effects are larger in females than in males. However, due to the power bias in the sex-combined GWAS, we have less power to detect loci with moderate degree of female bias than those with a similar degree of male bias. Thus, the estimated proportion of potential escape loci detected in a sex-combined analysis may be an underestimate because of the male-biased locus discovery in chrX. At a chromosome level the escape from XCI may be small, as it is reported to affect 15%–30% of genes,^34^ and a previous study had found limited effect of escape when considering the contribution of all X-linked complex trait loci.^4^

### Genotype and phenotype data in UK Biobank

We used genotype datasets from UKB^27^ (release version 3 of imputed genotype data) for all analyses in the study. The details on genotyping, quality control, and imputation have been described previously.^27^ Participants provided electronic signed consent at recruitment. Ethics approval for UKB was obtained from the North West Centre for Research Ethics Committee (11/NW/0382). All experiments were performed in accordance with relevant guidelines and regulations including the Declaration of Helsinki ethical principles for medical research.

The samples were included based on the following four criteria reported by the sample quality control file (“ukb_sqc_v2.txt.gz”) from UKB.

(1) Not an outlier for heterozygosity and missing rates (the “het.missing.outliers” column)
(2) Do not show putative sex chromosome aneuploidies (the “putative.sex.chromosome.aneuploidy” column)
(3) Reported sex matches with inferred sex (the “Submitted.Gender” and “Inferred.Gender” column)
(4) Were included in relatedness calculations (the “excluded.from.kinship.inference” column)

We restricted our analyses to unrelated white British, which were defined as those with a KING’s kinship coefficient less than the lower bound for the commonly used range to classify third-degree relatives (0.0442). We used the “in.white.British.ancestry.subset” column in the sample quality control file to define the white British. We removed individuals who had withdrawn from UKB by the time of this study.

All phenotypes were adjusted for males and females separately with appropriate covariates and inverse-normal transformed (Table S2), with values over six standard deviations from the mean removed as outliers prior to the normalization. For forced vital capacity and diastolic and systolic blood pressures, the means were taken for individuals with repeated measures. For individuals on blood pressure medications at baseline measurement (UKB field ID: 6153 and 6177), 15 mmHg and 10 mmHg were added to their measured values of systolic and diastolic blood pressures, respectively, following previous blood pressure analyses.^35^^,^^36^ UKB includes measurements for 34 blood and urine biomarkers. In this study, rheumatoid factor, estradiol, and microalbumin were excluded due to the large amount of missing data caused by the detection limits. For the remaining 31 biomarkers, we performed statin usage adjustment as described previously.^37^ In brief, we retrieved medication information from Treatment/medication (UKB field ID: 20003) and identified 1,296 individuals who were not on statin during the initial visit (years 2006–2010) but were on statin during their first repeat visit (years 2012–2013). A statin correction factor was calculated for each biomarker by taking the mean value of the ratio of on-statin measurement to pre-statin measurement. For 56,360 individuals who were taking statins upon enrollment, their biomarker measurements were divided by the statin correction factor to yield the adjusted values. The pre- and on-statin values were compared, and only biomarkers showing a significant difference (*p* value <0.05, paired Wilcoxon rank-sum test) were adjusted with the statin correction factors (Table S2).

For non-biomarker quantitative traits, we included traits with estimated autosomal $h^{2}$ ≥ 10% in sex-combined population in the UKB SNP-Heritability Browser (see web resources) that are available for both sexes. To avoid taking redundant traits (e.g., traits such as impedance of left and right legs), we performed a hierarchical clustering and identified 15 clusters (Figure S2) based on the correlation of adjusted and normalized values between these traits in the sex-combined population. Within each cluster, the trait with the highest $h^{2}$ estimated in the UKB SNP-Heritability Browser was selected for GWAS. In addition, for their medical relevance, we included diastolic blood pressure (clustered with systolic blood pressure) and body mass index (BMI, clustered with weight, waist, and hip circumferences).

### Genome-wide association analyses in UKB

In all analyses, the non-PAR genotypes were coded as {0,2} in males and {0,1,2} in females. Both sex-specific and sex-combined GWASs were performed with BOLT-LMM v.2.3.2^29^ for autosomes and chrX (both PAR and non-PAR). Directly genotyped variants (version 2) with MAF >0.01 and missingness <10% in autosomes and non-PAR chrX were used as the set of model SNPs in BOLT-LMM to estimate genetic relationship matrix and adjust the GWAS for confounding. GWAS statistics were calculated for imputed SNPs (version 3) with MAF >0.001 and imputation quality >0.7.

### Validation in FinnGen

The FinnGen data release 10 comprised 430,897 genotyped Finnish individuals. The detailed permits and biobank decisions are provided in the supplemental notes. The details on genotyping, quality control and imputation have been described previously.^28^ In brief, genotype imputation was performed with the SISu v.4.2 reference panel including 8,554 high-coverage whole-genome sequenced Finnish individuals for autosomes and non-PAR of chrX. We performed sex-specific GWASs for height, BMI, and weight with an average of 139,247 males and 154,408 females per trait using the REGENIE v.2.2.4 pipeline with similar covariates included as in the UKB GWASs (Table S2).

### Estimation of SNP heritability and effect-size distribution in chrX and autosomes

GENESIS^38^ is a likelihood-based approach for estimating effect-size distribution and heritability and by default only analyzes autosomal summary statistics, as only autosomal SNPs were included in the reference panel. To extend it to include chrX, we extracted the tagging SNPs and calculated the corresponding linkage disequilibrium (LD) scores in chrX as described in Zhang et al.^38^ In brief, we included HapMap3 SNPs with MAF ≥0.05 in the 1000 Genomes Project phase 3 study^39^ of 489 individuals of European origin^40^ for PAR region and 256 females of European origin^40^ for non-PAR as our reference panel for chrX. Here, the tagging SNPs for a GWAS SNP were defined as those in the reference panel that were within 1 Mb distance and had an estimated LD coefficient ($r^{2}$) with the GWAS marker above 0.1. We calculated the corresponding LD score for each GWAS marker by summing up the $r^{2}$ for all tagging SNPs using 1000 Genomes Project data of both sexes for PAR and only females for non-PAR. We adjusted the LD score for bias as in Zhang et al.^38^^,^^41^ and Gazal et al.^38^^,^^41^ In total, 38,231 X-linked common variants (439 in PAR and 37,792 in non-PAR) were included in the reference panel for GENESIS analysis.

We analyzed the sex-specific summary statistics from the GWAS for each trait with two-component model (referred as M2 in GENESIS) for the 38,231 common variants in chrX. We performed the analyses with ∼1.1 million common variants (MAF ≥0.05, excluding the major histocompatibility complex [MHC] region) in autosomes that was already included in GENESIS. GENESIS estimates the proportion of non-null-effect variants ($\pi_{c}$) and $h^{2}$ explained per causal variant ($\sigma^{2}$) that together define the effect-size distribution. The SNP heritability is defined as $h^{2}=M_{C}\sigma^{2}=M\pi_{C}\sigma^{2}$, with $M_{C}$ being the number of causal SNPs that is determined by $\pi_{c}$ and M, the total number of HapMap 3 SNPs (38,231 in chrX and in 1,070,777 in autosomes).

For comparison, we estimated SNP heritabilities of the 48 traits with LD score regression^41^ using only autosomal sex-specific summary statistics. Precomputed LD scores of European individuals in 1000 Genomes Project were used as reference consisting of ∼1.2 million variants in autosomes.^41^

We tested whether $h_{X}^{2}=0$ using the test statistic,

$$T=\frac{{\hat{h_{X}^{2}}}^{2}}{{\text{SE}}^{2}\left(\hat{h_{X}^{2}}\right)}\text{,}$$

where $\hat{h_{X}^{2}}$ is the GENESIS estimates of SNP heritabilities in chrX and $SE\left(\hat{h_{X}^{2}}\right)$ is the standard error (SE) of $\hat{h_{X}^{2}}$. The test statistic follows a $\chi^{2}$ distribution with 1° of freedom. The obtained *p* values and false discovery rate (FDR) (using Benjamini-Hochberg procedure) are reported in Table S3.

### Estimation of X chromosome influence

We estimated the contribution of chrX in complex trait genetics by defining the X chromosome influence (XI) as

$$\text{XI}=\frac{\hat{h_{X}^{2}}}{\hat{h_{A}^{2}}}\text{,}$$

where $\hat{h_{X}^{2}}$ and $\hat{h_{A}^{2}}$ are the GENESIS estimates of SNP heritabilities in chrX and autosomes, respectively.

The SE of XI was estimated as

$$\text{SE}=\frac{\hat{h_{X}^{2}}}{\hat{h_{A}^{2}}}\sqrt{\frac{{\text{SE}}^{2}\left(\hat{h_{X}^{2}}\right)}{{\left(\hat{h_{X}^{2}}\right)}^{2}}+\frac{{\text{SE}}^{2}\left(\hat{h_{A}^{2}}\right)}{{\left(\hat{h_{A}^{2}}\right)}^{2}}}\text{,}$$

where $\text{SE}\left(\hat{h_{X}^{2}}\right)$ and $\text{SE}\left(\hat{h_{A}^{2}}\right)$ are the corresponding SEs of $\hat{h_{X}^{2}}$ and $\hat{h_{A}^{2}}$ estimated with GENESIS.

We compared the observed XI to the XI predicted by the ratio (ϕ) of the number of variants in chrX to that in autosomes that contribute to heritability under two scenarios, X = AA and X = A (see Figure 1). When the active allele effects of chrX are 2-fold compared to autosomes (X = AA), we expected the XI to be 2ϕ in males, ϕ in females, and 3ϕ/2 (between-sex mean) in the sex-combined population. When the active allele effects are equal between chrX and autosomes (X = A), we expected the XI to be ϕ/2 in males, ϕ/4 in females, and 3ϕ/8 in the sex-combined population.

We approximated ϕ≈0.034 as the ratio of number of variants with MAF ≥0.01 in chrX to that in autosomes in the European population of the 1000 Genomes Project phase 3.^39^ We also included the results with ϕ, estimated based on the number of LD blocks present in chrX region and in autosomes for reference. The semi-LD-independent blocks were estimated using the LAVA partitioning algorithm^42^ using 263 female individuals of European ancestry of phase 3 of 1000 Genomes,^39^ resulting in 71 LD blocks in non-PAR and five LD blocks in PAR (Table S8). We performed the partitioning with the same parameters as had earlier been used for partitioning the autosomes (excluding the MHC region) into 2,479 LD blocks, that is, the default values of LAVA except that the minimum block size was set to 2,500 as in Werme et al.^42^ Based on the number of LD blocks, ϕ≈0.031.

### Identification of lead variants

Summary statistics of sex-specific GWASs were used to identify associated regions for each sex. For a SNP with a *p* value (non-infinitesimal model) below $5\times 10^{-8}$, a region of 0.5 Mb around the SNP was defined as the association region. Overlapping regions were merged and considered as the same association signal. The variants with the smallest *p* value within each region were considered as the lead variants. To compare effect sizes between autosomes and chrX, the effect-size estimates and the corresponding standard errors of variants within non-PAR were multiplied by 2 in both male- and female-specific analyses to estimate the active allele effects. This was done because the functionally hemizygous variants in non-PAR were analyzed as diploid under the coding scheme used for chrX.

We performed conditional analysis on UKB sex-combined GWASs for each associated region with FINEMAP v.1.4.^43^ The analysis was performed with default settings but allowing for a maximum of 30 causal SNPs (--n-causal-snps 30) and the posterior probability of a causal configuration to be zero if the absolute correlation of two SNPs is above 0.9 (--corr-config 0.9). We used LD computed from UKB genotype data with LDstore v.2.0 as recommended previously.^44^

### XCI scenarios analysis with sex-specific heritabilities

In theory, under full XCI (F-XCI), the $h_{X,m}^{2}$ is expected to be twice that of the $h_{X,f}^{2}$ in chrX^4^ (Figure 1). The absence of XCI (no XCI [N-XCI]) in females is expected to increase the female effect size 2-fold and hence result in male-to-female $h_{X}^{2}$ ratio of 0.5. Escape from XCI is expected to increase the $h_{X,f}^{2}$, yet to a much smaller degree than N-XCI, as escape affects only a fraction of the chrX loci and typically in a manner where the expression from the inactive X remains partially suppressed. To derive a meaningful male-to-female $h_{X}^{2}$ ratio for partial escape from XCI (E-XCI), we took the assumption of 25% of chrX loci undergoing escape. Further, following findings from gene-expression studies,^15^ where the expression from the inactive chrX is observed to be on average 33% of the expression from the active chrX, we assumed the effects from the inactive chrX to remain smaller than from the active chrX. To this end, we modeled the escape loci to follow approximately the relationship $\beta_{f}=\sqrt{2\cdot }\beta_{m}$ (Figure 1), i.e., a ratio $\sqrt{2}\approx 1.4$ of female-to-male effects (here we analyzed effects under {0,1,2} and {0,2} coding scheme, but the same relationship can be assumed for active allele effects, i.e., {0,0.5,1} and {0,1} coding scheme). Together, these assumptions translate to a male-to-female $h_{X}^{2}$ ratio for E-XCI at 1.75 (=2 × 75% + 1 × 25%).

We applied the “linemodels” package^45^ to the sex-specific $h^{2}$ estimates of 34 traits with non-zero $h_{X}^{2}$ in both sexes. We clustered the traits into three groups that were represented by line models, whose slopes were set to 2 (F-XCI), 1.75 (E-XCI), and 0.5 (N-XCI) for the chrX analysis and to 1 (F-XCI), 0.875 (E-XCI), and 0.25 (N-XCI) for the autosomal analysis, assuming the same relationship between male and female genetic effects as in the chrX for the different XCI scenarios. For all models, the initial values for the scale parameters were set as the larger standard deviations of the $h^{2}$ estimates across traits between male and female, the correlation parameters were fixed at 0.999, an equal prior probability across the models was assumed, and the correlation of male and female $h^{2}$ estimators was set to 0 because the samples were disjoint. The scale parameters were optimized in a two-step manner: first, we forced equal scales for all models by setting force.same.scales = TRUE in the line.models.optimize() function; second, we used the optimized scale parameters and estimated proportions of models as initial values in the line.models.optimize() function when we allowed different values for the scale parameters (force.same.scales = FALSE). Following the optimization of scale parameters for three models, we estimated the posterior probabilities in the three models separately for each trait with an equal prior probability assumed for each model. The analyses were performed separately for chrX and autosomal $h^{2}$ estimates.

### Four-component sex bias mixture model of genome-wide variants

To detect moderate sex-biased effects of variants across the genome, we used a mixture model with four components: null effect (M0), female-biased effect (M1), equal effect (M2), and male-biased effect (M3). The mixture model was constructed and fit in STAN (version 2.21.0).^46^ The distribution of each component was formulated with sex-specific summary statistics with effect size $\hat{\beta }$ and its $\hat{\text{SE}}$ scaled multiplicatively by $\sqrt{2f\left(1-f\right)}$, where f was MAF, so that, *a priori*, every variant explained similar phenotypic variance.

We denote by $\beta_{m}$ and $\beta_{f}$ the true male and female effects, respectively. Given the values of prior variance of the effect ($\sigma^{2})$ and a parameter α>1, the prior distributions of the components were

(1) M0: null effect, $\beta_{m}=\beta_{f}=0$;
(2) M1: female-biased effect, $\beta_{f}={\alpha \beta }_{m},\beta_{m}\;∼\;N\left(0,\sigma^{2}\right)$;
(3) M2: equal effect, $\beta_{m}=\beta_{f}\;∼\;N\left(0,\sigma^{2}\right)$; and
(4) M3: male-biased effect, $\beta_{m}={\alpha \beta }_{f},\beta_{f}\;∼\;N\left(0,\sigma^{2}\right)$.

Thus, $\sigma^{2}$ was assumed the same for each non-null component and its prior distribution was Uniform(0,1) (see supplemental notes for the choice of this prior). Additionally, the model includes parameter vector $\pi =\left(\pi_{0},\pi_{1},\pi_{2},\pi_{3}\right)$, where $\pi_{k}$ describes the proportion of variants belonging to component k. For π, we used Dirichlet(1/4, 1/4, 1/4, 1/4) distribution as the prior to not favor any component *a priori*.

This model, together with normally distributed effect-size estimates with known standard errors, leads to the following marginal distribution for the observed data (${\hat{\beta }}_{f},{\hat{\beta }}_{m},{\hat{\text{SE}}}_{f}^{2},{\hat{\text{SE}}}_{m}^{2})$:

$$\left[\begin{array}{c} {\hat{\beta }}_{f}\\ {\hat{\beta }}_{m} \end{array}\right]\;∼\;N\left(0,\left[\begin{array}{cc} {\hat{\text{SE}}}_{f}^{2} & 0\\ 0 & {\hat{\text{SE}}}_{m}^{2} \end{array}\right]+\sigma^{2}\Sigma_{k}\right)\text{,}$$

where for each non-null model the correlation between ${\hat{\beta }}_{f}$ and ${\hat{\beta }}_{m}$ was assumed to be 1:

$$\Sigma_{0}=\left[\begin{array}{cc} 0 & 0\\ 0 & 0 \end{array}\right],\Sigma_{1}=\left[\begin{array}{cc} \alpha^{2} & \alpha \\ \alpha & 1 \end{array}\right],\Sigma_{2}=\left[\begin{array}{cc} 1 & 1\\ 1 & 1 \end{array}\right],\Sigma_{3}=\left[\begin{array}{cc} 1 & \alpha \\ \alpha & \alpha^{2} \end{array}\right]\text{.}$$

In our analyses, we set $\alpha =\sqrt{2}$ because, in the female-biased component, we expected the effect of escape from XCI would result only in a moderate, clearly less than 2-fold increase in female effect compared to male effect.

Using this model, we estimated $\sigma^{2}$, the variance of the non-null effects, and $\pi_{k}$ values, the proportions of variants belonging to each component.

To identify variants with sex-biased effects, the estimated parameters were used to calculate a probability for variants to be assigned to a given component. For variant i, the probability $p_{i,k}$ for it to be in component k is^47^

$$p_{i,k}=\frac{{\hat{\pi }}_{k}N\left(\hat{\beta_{i}}:0,{\hat{S}}_{i}+\Sigma_{k}\right)}{\sum_{k}{\hat{\pi }}_{k}N\left(\hat{\beta_{i}}:0,{\hat{S}}_{i}+\Sigma_{k}\right)}\text{,}$$

where ${\hat{\pi }}_{k}$ is the posterior mean of proportion estimated by the model and ${\hat{\beta }}_{i}=\left[\begin{array}{c} {\hat{\beta }}_{i,f}\\ {\hat{\beta }}_{i,m} \end{array}\right],{\hat{S}}_{i}=\left[\begin{array}{cc} {\hat{\text{SE}}}_{i,f}^{2} & 0\\ 0 & {\hat{\text{SE}}}_{i,m}^{2} \end{array}\right]$. Variants were assigned to k component if the posterior probability $p_{i,k}>0.8$; otherwise, they were left “uncategorized.”

We focused on variants outside of the MHC region and excluded variants with missingness >1% and MAF <0.01 in both sexes in both autosomes and chrX, and Hardy-Weinberg disequilibrium test *p* value <$10^{-7}$ followed by LD pruning with PLINK 1.9^32^ using “--indep-pairwise 50 5 0.1” with genotype data from both sexes for autosomes and PAR and genotype data from females for non-PAR. This resulted in 4,380 variants in chrX and 152,091 variants in autosomes. For chrX, the analyses were performed using all 4,380 variants in chrX. For autosomes, six variants were sampled per LD group (total 1,693 LD groups) estimated in Europeans,^48^ resulting in 10,158 variants to allow efficient estimation of the parameters. All models were run with four chains, using 2,000 warm-up and 4,000 total iterations. Convergence was assessed using Rhat, which measures consistency of chains, and traits with parameter fits with Rhat greater than 1.01 were excluded.

## Results

### Contribution of chrX to complex traits

We first performed a sex-specific GWAS, using both autosomal and chrX, with BOLT-LMM^29^ in UKB for 48 quantitative traits (see details of traits selection in material and methods and trait information in Table S2). For validation purposes, we conducted similar association analyses for three of the UKB traits (height, BMI, and weight) in FinnGen^28^ (release 10).

We asked how much the additive genetic effects in chrX contribute to quantitative trait variation in the overall population. To this end, we estimated the sex-combined $h_{X}^{2}$ as the average of the male- and female-specific estimates from GENESIS,^38^ which we extended to allow the inclusion of chrX (see material and methods). Sex-specific data were used to avoid the power biases impacting the analyses of chrX variation in sex-combined data (see material and methods). For comparison, the same method was applied to autosomal data. We observed a clear role of chrX variation in most complex traits, as 45 out of the 48 analyzed UKB traits showed $h_{X}^{2}$ estimates significantly different from zero (FDR < 0.05), with height displaying the highest $h_{X}^{2}$ (UKB: 2.89% [SE = 0.30%]; FinnGen: 3.04% [SE = 0.31%]) (Table S3).

**Figure 2:**
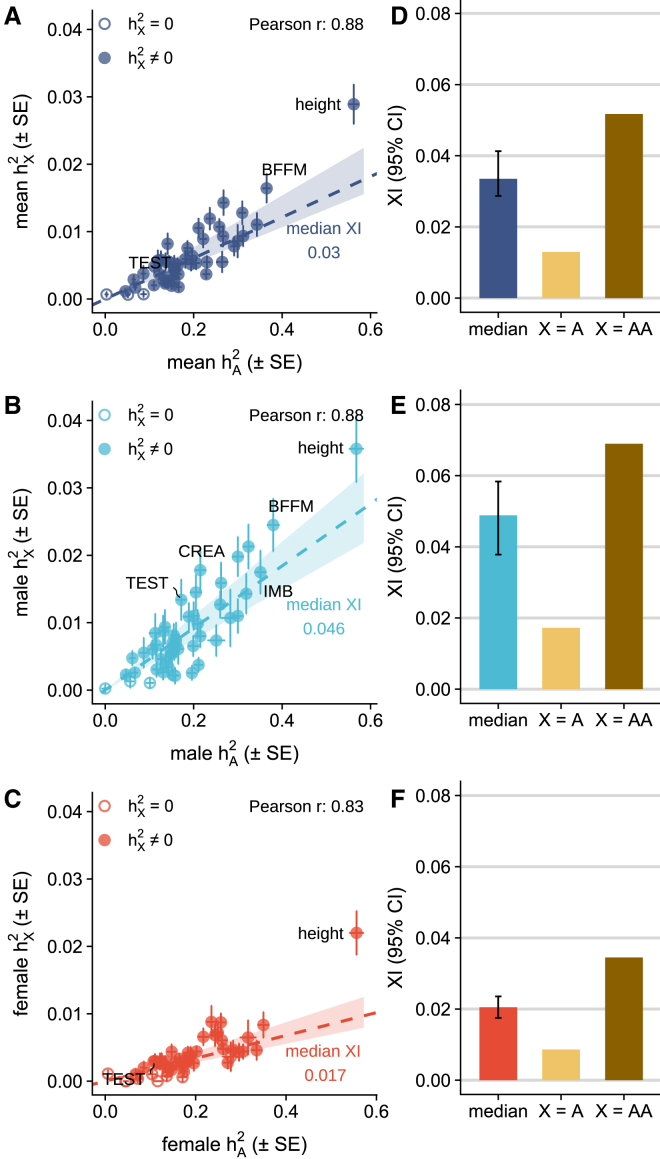
The contribution of chrX to complex trait genetics $h_{X}^{2}$ versus $h_{A}^{2}$ with error bars representing standard errors (A) in the overall population estimated as the average of sexes, (B) in males, and (C) in females. Dashed lines indicate the median XI, i.e., $h_{X}^{2}/h_{A}^{2}$, with shaded area indicating bootstrap 95% CI of the median. For 35 traits with non-zero $h_{X}^{2}$ in both sexes, the median XI (bootstrap 95% CI) is compared to the expected XI derived based on the proportion of common variants in chrX and assuming the genetic effect of one active X is equal to one copy of an autosome (X = A) or to a pair of autosomes (X = AA) (D) in the overall population, (E) in males, and (F) in females. Numeric results are reported in Table S3. BFFM, whole-body fat-free mass; IMB, impedance of body; CREA, creatinine; TEST, testosterone.

We next compared the estimated $h_{X}^{2}$ to the corresponding $h_{A}^{2}$ to understand the relative importance of chrX variation in complex traits. Across all the analyzed traits, $h_{X}^{2}$ tracked with $h_{A}^{2}$ (Pearson’s r = 0.88; Figure 2A) indicating the contribution from chrX to a complex trait is typically proportional to the autosomal contribution. To further quantify the role of chrX in quantitative trait variation, we defined the XI as the ratio of $h_{X}^{2}$ to $h_{A}^{2}$ and calculated this quantity separately for each trait. We observed a median XI of 0.03 (Figure 2A) suggesting that, in the overall population, chrX contributes to complex trait heritability an additional 3% of the contribution of autosomes.

Given the unique sex-dependent biology of chrX, we assessed how chrX contributes to complex trait variation differently between sexes by comparing the sex-specific $h^{2}$ estimates. As expected, given the chrX dosage difference between the sexes and XCI in females (see “complications and consequences of chrX biology in GWASs”), we observed, in general, higher $h_{X}^{2}$ in males compared to females (mean 0.88% [SD = 0.69%] versus 0.37% [SD = 0.35%]; *p* = $3.54\times 10^{-11}$, paired Wilcoxon rank-sum test), consistent with earlier reports.^4^^,^^5^ This higher $h_{X}^{2}$ in males is reflected also in the 2.3-fold greater number of X-linked genome-wide significant loci in males compared to females (Figure S3 and supplemental notes). In contrast, we observed no systematic sex difference in $h_{A}^{2}$ (*p* = 0.89, paired Wilcoxon rank-sum test), although a few traits (5/48), namely testosterone, diastolic blood pressure, urate, insulin growth factor-1, and waist-to-hip ratio, showed a significant sex difference in $h_{A}^{2}$ (FDR < 0.05) as reported previously^47^^,^^49^^,^^50^^,^^51^ (Table S3).

As expected, given the above results, a clear sex difference was also observed in XI, with a consistent pattern of greater XI in males compared to females (Figures 2B and 2C; median 0.046 versus 0.017; *p* = $3.91\times 10^{-13}$, paired Wilcoxon rank-sum test). Interestingly, diastolic and systolic blood pressures were exceptions to this pattern, with greater XI in females than in males (diastolic blood pressure: 0.018 [SE = 0.0036] versus 0.013 [SE = 0.0058]; systolic blood pressure: 0.029 [SE = 0.0066] versus 0.018 [SE = 0.0053]). Together, however, these observations exemplify the greater relative importance of the chrX variation in males compared to females arising from the impact of the ploidy difference and chromosome-wide inactivation in females.

### Interpreting XI through the lens of dosage compensation

To provide insights into the potential dosage compensation between chrX and autosomes, we compared the above XI results to theoretical expectations of chrX-to-autosomes relationship under two scenarios. In the first scenario (X = AA), one active chrX is equivalent to a pair of autosomes, with 2-fold active allele effects in chrX compared to autosomes. In the second scenario (X = A), one active chrX is equivalent to a single autosome, with equal active allele effects between chrX and autosomes (see Figure 1 and “complications and consequences of chrX biology in GWASs”). For these theoretical expectations, we assumed that the allelic effects are small and uniformly distributed along the genome, and the expected values were computed based on the proportion of common variants (chrX contains ∼3.4% of the number of common variants in autosomes; see “estimation of X chromosome influence”).

Focusing on the 35 traits with non-zero $h_{X}^{2}$ in both sexes, we observed that the medians of XI differed significantly (*p* value <0.05 based on bootstrap 95% confidence interval [CI]) from the expected values under both scenarios (Figures 2D–2F). In the overall population, the observed median XI (0.034, bootstrap 95% CI 0.029–0.041) was 1.5-fold lower than expected under X = AA (0.052) and 2.6-fold greater than expected under X = A (0.013) (Figure 2D). A similar degree of difference was observed in the sex-specific data (Figures 2E and 2F). Similar patterns were also observed with expected values derived based on the number of LD blocks, of which chrX contains 3.1% of those in the autosomes (Figure S4).

Dissection of $h^{2}$ into the proportion of causal variants $(\pi_{c}$, which estimates polygenicity) and per-SNP-$h^{2}$ ($\sigma^{2}$, which estimates the magnitude of non-zero effects) suggested the mismatch between the observed XI and assumptions under the dosage-compensation models arises from the effect sizes rather than from systematic differences in the polygenicity between chrX and the autosomes. While the estimated polygenicity varied greatly between chrX and autosomes (ratio of $\pi_{c}$ from chrX and autosomes ranges between 0.3 and 4 for most traits) (Figure S5), the observed medians of $\sigma_{X}^{2}/\sigma_{A}^{2}$ were again significantly lower than expected under X = AA (1.5- and 2.4-fold smaller than the expected in males and females, respectively) and higher than expected under X = A (2.5- and 1.7-fold greater than the expected in males and females, respectively) (Figure S6), closely mirroring the results from the XI comparisons. As heritability is a function of the squared effect size, the observed enriched per-SNP-$h^{2}$ in chrX under X = A translates to a median of 1.6- and 1.3-fold larger active allele effects in chrX than in autosomes in males and females, respectively.

These observations therefore suggest that, although there is only a single active copy of chrX in both sexes owing to the hemizygosity of men and XCI in females, the single active copy of chrX contributes to complex trait heritability more than a single autosomal copy with similar amount of genetic variation but less than two such autosomal copies.

### Comparison of active allelic effects between chrX and autosomes

**Figure 3:**
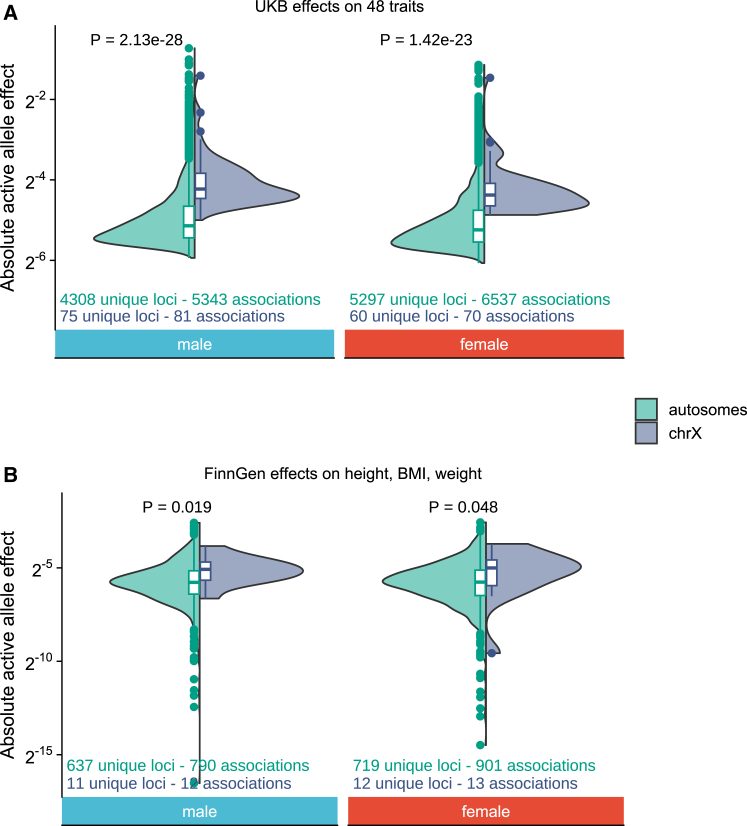
Comparison of active allele effects between chrX and autosomes Comparison of active allele effect size (a) between autosomes and chrX for sex-specific trait-associated variants identified in UKB (A) with a estimated in UKB for all 48 traits and (B) with a estimated in FinnGen for height, BMI, and weight. The male GWAS in non-PAR has been downsampled by half to have similar statistical power as in autosomal GWAS. *p* values for the difference between autosomes and chrX (Wilcox rank-sum test) are indicated on the top. Numerical values are reported in Table S7.

To formally test for the differences in effect sizes, as suggested by the above results, we assessed the chrX-to-autosome differences in allelic effect estimates across the studied traits. To this end, we compared the effect sizes per active allele (see material and methods) of lead variants in chrX ($a_{X}$) to those of autosomes ($a_{A}$) identified from the male and female GWASs, where the males were downsampled by half in chrX association analyses to achieve similar power as in the autosomes (see “complications and consequences of chrX biology in GWASs”). We observed that the medians of $a_{X}$ were 1.9- and 1.8-fold higher in chrX than that of $a_{A}$ in males and females, respectively (0.054 versus 0.028 in males, 0.048 versus 0.026 in females; *p* = $2.13\times 10^{-28}$ and $1.42\times 10^{-23}$ for males and females, respectively, Wilcoxon rank-sum test) (Figure 3A), a pattern not influenced by pleiotropic loci (Figure S7A). To eliminate the effect of the “winner’s curse,” we further compared the effects estimated using FinnGen data for the height-, BMI-, and weight-associated variants identified from the UKB data. Although limited by the numbers of variants in chrX, the difference between the chrX and autosomes remained, with the medians of $a_{X}$ being 1.6- and 1.7-fold higher than the medians of $a_{A}$, in males and females, respectively (Figure 3B).

We assessed whether these observations were explained by differences in MAF, functional consequences, or pathogenicity of variants between chrX and autosomes. We observed that chrX overall has slightly higher MAF, fewer regulatory and coding regions, and fewer pathogenic variants relative to autosomes. However, we observed systematically larger $a_{X}$ than $a_{A}$ independent of variant frequency or consequence. An exception was the most constrained regions, in which variants are rare in chrX and an upper bound may be imposed on the active allele effects by negative selection (Figures S8–S10 and supplemental notes).

Taken together, these observations suggest that, overall, common variants in chrX have larger active allele effects compared to the autosomes, which likely explains the higher XI compared to the expected under X = A that only one functional copy of chrX is present in each cell.

### Insight into XCI escape through sex-specific heritability comparison

Our earlier results of $h_{X}^{2}$ indicated a clear sex difference consistent with the presence of XCI (Figures 2B and 2C). To further understand the completeness of XCI through complex trait genetics, we compared how different XCI scenarios explain the observed relationship of male and female $h_{X}^{2}$, partly following the approach of Sidorenko et al.^4^ To this end, we applied a Bayesian approach^45^ to cluster the traits to the three XCI models—F-XCI (expected male-to-female $h_{X}^{2}$ ratio = 2), N-XCI (expected ratio at 0.5), and E-XCI (expected ratio at 1.75)—that accounts for a scenario where 25% of the chrX loci partially escape from XCI (see “XCI scenarios analysis with sex-specific heritabilities”). While such a chromosome-level heritability comparison does not allow the identification of individual loci impacted by escape, this approach can inform on which traits are under the influence of the collective effect of escape.

**Figure 4:**
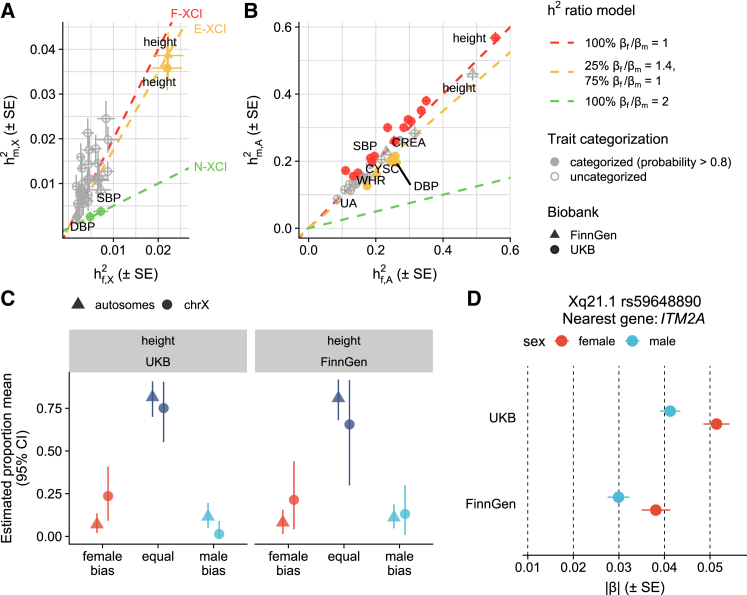
Comparison of male and female $h^{2}$ and estimated SNP effects (A and B) Comparison of male and female $h^{2}$ with error bars representing standard errors in (A) chrX and (B) autosomes for each trait and clustering based on theoretical XCI scenarios. The red, yellow, and green dashed lines indicate expectation under full XCI (F-XCI), 25% escape from XCI (E-XCI), and no XCI (N-XCI), respectively. Colored points belong to the cluster with probability >0.80. Numeric values are reported in Table S9. (C) Estimated proportion of variants with female-biased, equal, and male-biased effect on height in UKB and FinnGen. Error bars represent 95% confidence intervals. Numeric values are reported in Table S10. (D) Sex-specific effects of rs59648890 variant near *ITM2A* (integral membrane protein 2A) on height in UKB and FinnGen, with error bars representing standard errors. DBP, diastolic blood pressure; SBP, systolic blood pressure; CREA, creatinine; UA, urate; WHR, waist-to-hip ratio; CYSC, cystatin C.

Using a posterior probability threshold of 0.80, for most of the traits we were unable to distinguish between the F-XCI and the E-XCI models (Figure 4A). For instance, forced vital capacity had F-XCI posterior probability of 0.41 and E-XCI posterior probability of 0.59. This uncertainty in assignments reflects both the subtle difference between the expectations of the F-XCI and E-XCI models and fairly small heritabilities in the chrX for most of the traits studied. However, diastolic and systolic blood pressures clustered to the N-XCI model with posterior probabilities of 0.98 (Figure 4A). For these blood pressure traits, we also observed larger $h_{A}^{2}$ in females than in males (Figure 4B), as reported previously,^52^ suggesting that the sex differences in $h_{X}^{2}$ are unlikely to be explained by the lack of or escape from XCI alone but may be attributable to other factors such as hormonal influences.^53^ The only trait that was best explained by the partial escape from XCI model was height (E-XCI posterior probability = 0.99) (Figure 4A), a trait that we found highly heritable in chrX (Figures 2A–2C). A similar sex difference in $h_{X}^{2}$ for height was also detected in FinnGen (E-XCI posterior probability = 1.00) (Figure 4A), but no sex difference was seen in autosomal data for height in either of the datasets (Figure 4B). The finding of female-enriched $h_{X}^{2}$ points to the potential role of chrX loci escaping from XCI in human height.

### Comparison of sex-biased effects between chrX and autosomes

To further elucidate the potential sex-dependent genetic architecture in chrX, we asked whether the proportions of variants with either female-biased or male-biased effects differ between chrX and autosomes. To this end, we applied a mixture model to sex-specific summary statistics to estimate the proportion of genetic effects with female or male bias (see “four-component sex bias mixture model of genome-wide variants”). The mixture model contains four components to capture the following types of variants: no effect on the trait in either sex, equal non-null effects in males and females, female-biased effects ($\left|\beta_{f}\right|>\left|\beta_{m}\right|)$, and male-biased effects ($\left|\beta_{m}\right|>\left|\beta_{f}\right|)$.

Focusing on the proportions of variants with non-null effects, we observed that, compared to autosomes, there are proportionally fewer associations with equal genetic effects between sexes in chrX (*p* = $9.97\times 10^{-8}$, paired Wilcoxon rank-sum test of point estimates; Figure S11), pointing to unique sex-biased characteristics of chrX. This result was driven by a greater fraction in male-biased effects (*p* = $4.46\times 10^{-6}$, paired Wilcoxon rank-sum test of point estimates) rather than female-biased effects (*p* = 0.07, paired Wilcoxon rank-sum test of point estimates) in chrX. The enrichment of male-biased effects may be largely attributable to the pleiotropic male-specific effects of regions in chrX associated with testosterone, a trait known for enriched male-specific effects in chrX^47^^,^^54^ (see supplemental notes). Interestingly, for waist-to-hip ratio, a trait known for largely female-biased genetic effects in the autosomes,^55^ the majority of non-null variants were expectedly estimated to be female biased in autosomes (78.8% [95% CI: 51.1%–98.4%]); however, in chrX, a considerably smaller fraction, 16.8% (95% CI: 0%–67.0%) of non-null variants, were estimated be in the female-biased component (see supplemental notes).

Echoing the results of XCI analysis on male and female $h^{2}$ comparison on height, we observed a greater proportion of non-null variants with female-biased effects in chrX compared to autosomes for height (23.6% [95% CI: 9.57%–40.4%] versus 6.99% [95% CI: 2.35%–13.1%]) (Figure 4C). For validation, we performed the same analysis in FinnGen, where we observed the same pattern of an enrichment of female-biased effects among the non-null variants in chrX (21.4% [95% CI: 4.47%–43.7%] versus 8.09% [95% CI: 1.86%–15.2%]) (Figure 4C).

To pinpoint individual loci driving the observed female bias in chrX for height, we computed the posterior probability of each component for the genome-wide significant height-associated variants identified in sex-combined conditional analysis (*n* = 73). We identified eight lead variants as female biased (posterior probability >0.80); however, only one variant was replicated in FinnGen as a female-biased variant (Figure S12A). This highlights the poor consistency of sex differences across biobanks despite the highly reproducible genetic effects (Figures S12 and S13; supplemental notes). The replicated variant, rs59648890, locates 33 kb upstream of *ITM2A* (integral membrane protein 2A, a gene involved in cartilage development) (Figure 4D), confirming our earlier findings in a smaller Finnish sample.^7^ Supporting the SNP level finding, the male-to-female ratio of local $h^{2}$ at the LD block containing the *ITM2A* region (X: 77,844,781–80,093,260) was also smaller than expected under F-XCI at 2 ($h_{f}^{2}$ = 0.41% and $h_{m}^{2}$ = 0.53% in UKB; $h_{f}^{2}$ = 0.55% and $h_{m}^{2}$ = 0.98% in FinnGen). The moderate female-biased effect (1.2 times greater in females than in males in UKB and 1.3 times greater in FinnGen) at the locus is aligned with partial escape from XCI.

## Discussion

ChrX has remained understudied in GWASs, largely owing to the distinct challenges it poses for the analysis and interpretation of genetic associations. We set out to provide a thorough understanding on how the chromosome and its unique biology contribute to complex traits. Through analyzing large-scale biobank data across a broad panel of quantitative traits, we demonstrated that chrX hosts complex trait heritability and loci proportional to the contribution of autosomes. Our findings further support the presence of near-full XCI,^4^ the dosage compensation between XY males and XX females, and highlight the relevance of this process for the sex-specific contributions of the chromosome for complex traits. Our results also mirror the dosage-compensation mechanism between chrX and autosomes through X upregulation proposed by Ohno,^11^ whereby the contributions between chrX and autosomes are balanced through systematically larger chrX effect sizes per single active copy of the chromosome.

Across the 48 complex traits investigated, we found that, in the overall population, the chrX $h^{2}$ equated to approximately 3% of the autosomal $h^{2}$. As such, the contribution of chrX to complex trait variation is typically less than that of the chromosome size but is in line with the proportion of genetic variants in the chromosome, which is smaller than that of a similar-sized autosome. Considering that due to XCI only one copy of chrX is active in each sex, the observed XI, however, suggests a greater role for chrX than expected under X = A but smaller than expected under X = AA. We attribute this finding to the systematically larger, but less than 2-fold, active allele effects in chrX compared to autosomes. As such, the finding appears to parallel Ohno’s dosage-compensation hypothesis on a global transcriptional X upregulation to account for the dosage difference between a single active chrX and pairs of autosomes. While less studied than XCI, X upregulation has been shown to be present but partial through gene-expression analyses,^18^^,^^19^^,^^20^^,^^21^^,^^22^^,^^23^ proposed to occur through more frequent transcriptional bursting from chrX.^22^ Interestingly, our estimate of the active allele effect-size difference in complex trait genetic associations (∼1.6-fold) is close to the estimated degree of transcriptional X upregulation (∼1.4-fold).^22^ However, whether these two phenomena share a mechanistic basis warrants further investigations.

The observed average doubled contribution of chrX to phenotypic variation in males, echoing observations from other complex traits,^4^^,^^5^ is consistent with the joint effects of male hemizygosity and XCI and aligns with the established male preponderance for X-linked disorders where random XCI in females confers protection. The sex difference in chrX heritability is, however, unlikely to be reflected significantly in the genome-wide trait heritability estimates in the current sample sizes owing to chrX typically contributing only a few percentage points of the overall heritability. Further, it follows, given the greater role of chrX variation in males, that additive effects in chrX are unlikely to explain female biases in complex phenotypes.

Genetic data from chrX could theoretically be used to identify escape regions and traits being impacted, yet the anticipated subtle changes on genetic effects due to escape render such assessments highly challenging even in biobank-scale datasets like ours. We found plausible evidence consistent with partial escape only in height, a trait where we had the largest power for the assessment. After replication, we could pinpoint plausibly the *ITM2A* locus not subject to full XCI. The *ITM2A* locus has been reported previously to be associated with height, but evidence for escape is interpreted differently.^4^^,^^7^ Further validation and mechanistic dissection of this locus is warranted. Overall, it is possible that the proposed contribution of escape from XCI to phenotypic sex differences acts via mechanisms other than through direct locus-specific effects on phenotypes.

**Figure 5:**
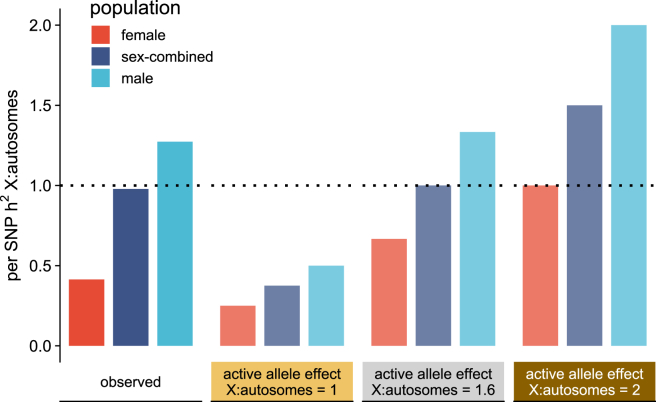
Model of how XI is optimally balanced in the population Illustration of chrX-to-autosomes per-SNP-$h^{2}$ ratio in male, female, and overall populations assuming full XCI for observed data (medians) in solid colors and for three theoretical chrX-to-autosomes active allele effect ratios shown in corresponding colors with reduced opacity. The observed per-SNP-$h^{2}$ in the sex-combined population is estimated as the mean across males and females. The dotted line indicates when the per-SNP-$h^{2}$ is equal between chrX and autosomes in the sex-combined population.

In the light of these findings, we propose that the two dosage-compensation mechanisms act in concert to optimally balance the role of chrX in the population. Owing to nearly full XCI, the per-SNP-$h^{2}$ is about twice as high in males as in females and resides in between these two estimates in the overall population. Given this, under equal active allele effects between chrX and autosomes, the per-SNP-$h^{2}$ in chrX would remain at relatively low level compared to autosomes in both sexes, so the contribution of chrX to complex traits would be much lower than expected by the amount of genetic variation in chrX (Figure 5, scenario X:autosomes = 1). Two-fold active allele effects in chrX would, however, increase the per-SNP-$h^{2}$ to a high level in males, where a possible upper bound may be imposed by negative selection on the hemizygous chrX in males (Figure 5, scenario X:autosomes = 2). A “partial upregulation” of chrX, with 1.6-fold larger active allele effects in chrX compared to autosomes, a scenario closely matching our observations, would balance out the sex difference in per-SNP-$h^{2}$ in the overall population, resulting in comparable per-SNP-$h^{2}$ between chrX and autosomes and the contribution of chrX to complex trait genetics on par with that of the autosomes (Figure 5, scenario X:autosomes = 1.6 and observed).

Our assessments in this study focused on the role of additive genetic variation in chrX. While additive effects are the primary mode of heritability in autosomes,^56^ the unique characteristics of chrX can make other types of effects more relevant. For instance, X-linked deleterious alleles that are known to play a role in rare diseases often affect females in a recessive manner. Further, skewed XCI, which changes the heterozygous dosage in females to homozygous, may be particularly relevant for higher-impact variation, and it has been observed at higher frequencies among individuals with autoimmune diseases.^57^^,^^58^ Also, our assessments included only quantitative traits, and different dynamics may be expected when extending chrX analyses to complex diseases.

Our primary studies were performed in UKB, which is a volunteer-based study with evidence for sex-differential participation bias in autosomes.^59^ Thus, where possible, we set out to validate our findings in FinnGen, a dataset with more passive participation design. While we observed the same female-biased pattern on the chromosome level for height, the sex-biased effects for individual loci were poorly replicated across biobanks, highlighting the broader challenges associated with the detection of gene-by-environment interactions.^60^^,^^61^

As most GWAS tools nowadays support the analysis of chrX, the inclusion of chrX, as shown in our study, offers a possibility to uncover new biology and trait $h^{2}$. Although the underlying assumptions regarding dosage compensation in chrX analyses may not be highly relevant for locus discovery, these matter for the interpretation of the relationship of male to female and chrX to autosome effects (see “complications and consequences of chrX biology in GWASs”). Further, while sex differences in complex trait genetic architecture are typically modest in chrX, one should nevertheless be aware of the power difference between the sexes that bias the detection toward male-biased effects when the variant selection is based on a significance threshold. The potential effects of escape, though shown here to be limited at the currently available sample sizes, may become evident as sample sizes in GWASs continue to grow.

Taken together, our work shows that in addition to providing new complex trait associations, GWAS data on chrX provides possibilities to delve into the unique biology of this chromosome.

## Data and code availability

Male and female GWAS summary statistics are available at: https://doi.org/10.5281/zenodo.15148429 and https://doi.org/10.5281/zenodo.15125257, respectively. Sex-combined GWAS summary statistics on height are available at https://doi.org/10.5281/zenodo.15131077. The code used in this study is available at https://github.com/yufugen/DC_GWAS.

## Acknowledgments

We greatly thank all UK Biobank and FinnGen participants as well as the principal investigators, laboratory personnel, and data-management teams behind these efforts. The research has been conducted using the UK Biobank Resource under application number 22627. Full FinnGen funders and FinnGen acknowledgments are provided in the supplemental acknowledgments. This work was financially supported by the 10.13039/100007797University of Helsinki Doctoral Program in Population Health (Y.F.), the 10.13039/501100002341Research Council of Finland (315589 and 320129 to T.T. and 338507, 336825, and 352795 to M.P.), the 10.13039/100015735HiLIFE Fellows Program (T.T.), and 10.13039/501100006306Sigrid Jusélius Foundation (T.T. and M.P.). The graphical abstract (https://BioRender.com/r43p126) and Figure 1 (https://BioRender.com/q91s752) were created with BioRender.

## Author contributions

Conceptualization, T.T., M.P., and Y.F.; acquisition, analysis, and interpretation of data, T.T., M.P., Y.F., A.K., and S.R.; drafting of the manuscript, Y.F. and T.T.; critical revision and editing of the manuscript, T.T., M.P., and Y.F.; visualization, Y.F.; supervision, T.T. and M.P.

## Declaration of interests

A.K. is a current employee of Nightingale Health Plc.

## Web resources

1000 Genomes Project phase 3, https://ftp.1000genomes.ebi.ac.uk/vol1/ftp/release/20130502/
BOLT-LMM, https://alkesgroup.broadinstitute.org/BOLT-LMM/BOLT-LMM_manual.html
FINEMAP, http://www.christianbenner.com/
GENESIS, https://github.com/yandorazhang/GENESIS
HapMap phase 3, ftp://ftp.ncbi.nlm.nih.gov/hapmap/phase_3
LAVA partitioning algorithm, https://github.com/cadeleeuw/lava-partitioning
LD score regression, https://github.com/bulik/ldsc
linemodels, https://github.com/mjpirinen/linemodels
PLINK 1.9, www.cog-genomics.org/plink/1.9/
REGENIE v.2.2.4 pipeline, https://github.com/FINNGEN/regenie-pipelines
UKB SNP-Heritability Browser, https://nealelab.github.io/UKBB_ldsc/h2_browser.html

## Supplemental information

Document S1. Figures S1–S20, Tables S1, S6, S8, S9, S11, and S12, supplemental notes, and supplemental acknowledgments

Data S1. Tables S2–S5, S7, S10, S13, and S14

Document S2. Article plus supplemental information

## References

[1] Sollis E., Mosaku A., Abid A., Buniello A., Cerezo M., Gil L., Groza T., Güneş O., Hall P., Hayhurst J. (2023). The NHGRI-EBI GWAS Catalog: knowledgebase and deposition resource. Nucleic Acids Res..

[2] Sun L., Wang Z., Lu T., Manolio T.A., Paterson A.D. (2023). eXclusionarY: 10 years later, where are the sex chromosomes in GWASs?. Am. J. Hum. Genet..

[3] Scholz M., Horn K., Pott J., Wuttke M., Kühnapfel A., Nasr M.K., Kirsten H., Li Y., Hoppmann A., Gorski M. (2024). X-chromosome and kidney function: evidence from a multi-trait genetic analysis of 908,697 individuals reveals sex-specific and sex-differential findings in genes regulated by androgen response elements. Nat. Commun..

[4] Sidorenko J., Kassam I., Kemper K.E., Zeng J., Lloyd-Jones L.R., Montgomery G.W., Gibson G., Metspalu A., Esko T., Yang J. (2019). The effect of X-linked dosage compensation on complex trait variation. Nat. Commun..

[5] Mallard T.T., Liu S., Seidlitz J., Ma Z., Moraczewski D., Thomas A., Raznahan A. (2021). X-chromosome influences on neuroanatomical variation in humans. Nat. Neurosci..

[6] Sinnott-Armstrong N., Naqvi S., Rivas M., Pritchard J.K. (2021). GWAS of three molecular traits highlights core genes and pathways alongside a highly polygenic background. Elife.

[7] Tukiainen T., Pirinen M., Sarin A.-P., Ladenvall C., Kettunen J., Lehtimäki T., Lokki M.-L., Perola M., Sinisalo J., Vlachopoulou E. (2014). Chromosome X-Wide Association Study Identifies Loci for Fasting Insulin and Height and Evidence for Incomplete Dosage Compensation. PLoS Genet..

[8] Natarajan P., Pampana A., Graham S.E., Ruotsalainen S.E., Perry J.A., de Vries P.S., Broome J.G., Pirruccello J.P., Honigberg M.C., Aragam K. (2021). Chromosome Xq23 is associated with lower atherogenic lipid concentrations and favorable cardiometabolic indices. Nat. Commun..

[9] Mendes M., Chen D.Z., Engchuan W., Leal T.P., Thiruvahindrapuram B., Trost B., Howe J.L., Pellecchia G., Nalpathamkalam T., Alexandrova R. (2025). Chromosome X-wide common variant association study in autism spectrum disorder. Am. J. Hum. Genet..

[10] Wise A.L., Gyi L., Manolio T.A. (2013). eXclusion: Toward Integrating the X Chromosome in Genome-wide Association Analyses. Am. J. Hum. Genet..

[11] Ohno S. (1967).

[12] Lyon M.F. (1961). Gene Action in the X-chromosome of the Mouse (Mus musculus L.). Nature.

[13] Lee J.T. (2011). Gracefully ageing at 50, X-chromosome inactivation becomes a paradigm for RNA and chromatin control. Nat. Rev. Mol. Cell Biol..

[14] Johnston C.M., Lovell F.L., Leongamornlert D.A., Stranger B.E., Dermitzakis E.T., Ross M.T. (2008). Large-Scale Population Study of Human Cell Lines Indicates that Dosage Compensation Is Virtually Complete. PLoS Genet..

[15] Tukiainen T., Villani A.-C., Yen A., Rivas M.A., Marshall J.L., Satija R., Aguirre M., Gauthier L., Fleharty M., Kirby A. (2017). Landscape of X chromosome inactivation across human tissues. Nature.

[16] Carrel L., Willard H.F. (2005). X-inactivation profile reveals extensive variability in X-linked gene expression in females. Nature.

[17] Cotton A.M., Ge B., Light N., Adoue V., Pastinen T., Brown C.J. (2013). Analysis of expressed SNPs identifies variable extents of expression from the human inactive X chromosome. Genome Biol..

[18] Nguyen D.K., Disteche C.M. (2006). Dosage compensation of the active X chromosome in mammals. Nat. Genet..

[19] Lin H., Halsall J.A., Antczak P., O’Neill L.P., Falciani F., Turner B.M. (2011). Relative overexpression of X-linked genes in mouse embryonic stem cells is consistent with Ohno’s hypothesis. Nat. Genet..

[20] Deng X., Hiatt J.B., Nguyen D.K., Ercan S., Sturgill D., Hillier L.W., Schlesinger F., Davis C.A., Reinke V.J., Gingeras T.R. (2011). Evidence for compensatory upregulation of expressed X-linked genes in mammals, Caenorhabditis elegans and Drosophila melanogaster. Nat. Genet..

[21] Pessia E., Makino T., Bailly-Bechet M., McLysaght A., Marais G.A.B. (2012). Mammalian X chromosome inactivation evolved as a dosage-compensation mechanism for dosage-sensitive genes on the X chromosome. Proc. Natl. Acad. Sci. USA.

[22] Larsson A.J.M., Coucoravas C., Sandberg R., Reinius B. (2019). X-chromosome upregulation is driven by increased burst frequency. Nat. Struct. Mol. Biol..

[23] Lentini A., Cheng H., Noble J.C., Papanicolaou N., Coucoravas C., Andrews N., Deng Q., Enge M., Reinius B. (2022). Elastic dosage compensation by X-chromosome upregulation. Nat. Commun..

[24] Kent J.W., Dyer T.D., Blangero J. (2005). Estimating the additive genetic effect of the X chromosome. Genet. Epidemiol..

[25] Tomofuji Y., Edahiro R., Sonehara K., Shirai Y., Kock K.H., Wang Q.S., Namba S., Moody J., Ando Y., Suzuki A. (2024). Quantification of escape from X chromosome inactivation with single-cell omics data reveals heterogeneity across cell types and tissues. Cell Genomics.

[26] Kukurba K.R., Parsana P., Balliu B., Smith K.S., Zappala Z., Knowles D.A., Favé M.-J., Davis J.R., Li X., Zhu X. (2016). Impact of the X Chromosome and sex on regulatory variation. Genome Res..

[27] Bycroft C., Freeman C., Petkova D., Band G., Elliott L.T., Sharp K., Motyer A., Vukcevic D., Delaneau O., O’Connell J. (2018). The UK Biobank resource with deep phenotyping and genomic data. Nature.

[28] Kurki M.I., Karjalainen J., Palta P., Sipilä T.P., Kristiansson K., Donner K.M., Reeve M.P., Laivuori H., Aavikko M., Kaunisto M.A. (2023). FinnGen provides genetic insights from a well-phenotyped isolated population. Nature.

[29] Loh P.-R., Kichaev G., Gazal S., Schoech A.P., Price A.L. (2018). Mixed-model association for biobank-scale datasets. Nat. Genet..

[30] Mbatchou J., Barnard L., Backman J., Marcketta A., Kosmicki J.A., Ziyatdinov A., Benner C., O’Dushlaine C., Barber M., Boutkov B. (2021). Computationally efficient whole-genome regression for quantitative and binary traits. Nat. Genet..

[31] Zhou W., Nielsen J.B., Fritsche L.G., Dey R., Gabrielsen M.E., Wolford B.N., LeFaive J., VandeHaar P., Gagliano S.A., Gifford A. (2018). Efficiently controlling for case-control imbalance and sample relatedness in large-scale genetic association studies. Nat. Genet..

[32] Chang C.C., Chow C.C., Tellier L.C., Vattikuti S., Purcell S.M., Lee J.J. (2015). Second-generation PLINK: rising to the challenge of larger and richer datasets. GigaScience.

[33] Clayton D. (2008). Testing for association on the X chromosome. Biostatistics.

[34] Posynick B.J., Brown C.J. (2019). Escape From X-Chromosome Inactivation: An Evolutionary Perspective. Front. Cell Dev. Biol..

[35] Tobin M.D., Sheehan N.A., Scurrah K.J., Burton P.R. (2005). Adjusting for treatment effects in studies of quantitative traits: antihypertensive therapy and systolic blood pressure. Stat. Med..

[36] Evangelou E., Warren H.R., Mosen-Ansorena D., Mifsud B., Pazoki R., Gao H., Ntritsos G., Dimou N., Cabrera C.P., Karaman I. (2018). Genetic analysis of over 1 million people identifies 535 new loci associated with blood pressure traits. Nat. Genet..

[37] Sinnott-Armstrong N., Tanigawa Y., Amar D., Mars N.J., Aguirre M., Venkataraman G.R., Wainberg M., Ollila H.M., Pirruccello J.P., Qian J. (2019). Genetics of 38 blood and urine biomarkers in the UK Biobank. bioRxiv.

[38] Zhang Y., Qi G., Park J.-H., Chatterjee N. (2018). Estimation of complex effect-size distributions using summary-level statistics from genome-wide association studies across 32 complex traits. Nat. Genet..

[39] Auton A., Abecasis G.R., Altshuler D.M., Durbin R.M., Abecasis G.R., Bentley D.R., Chakravarti A., Clark A.G., Donnelly P., Eichler E.E. (2015). A global reference for human genetic variation. Nature.

[40] Gazal S., Sahbatou M., Babron M.-C., Génin E., Leutenegger A.-L. (2015). High level of inbreeding in final phase of 1000 Genomes Project. Sci. Rep..

[41] Bulik-Sullivan B.K., Loh P.-R., Finucane H.K., Ripke S., Yang J., Patterson N., Daly M.J., Price A.L., Neale B.M., Schizophrenia Working Group of the Psychiatric Genomics Consortium (2015). LD Score regression distinguishes confounding from polygenicity in genome-wide association studies. Nat. Genet..

[42] Werme J., van der Sluis S., Posthuma D., de Leeuw C.A. (2022). An integrated framework for local genetic correlation analysis. Nat. Genet..

[43] Benner C., Spencer C.C.A., Havulinna A.S., Salomaa V., Ripatti S., Pirinen M. (2016). FINEMAP: efficient variable selection using summary data from genome-wide association studies. Bioinformatics.

[44] Benner C., Havulinna A.S., Järvelin M.-R., Salomaa V., Ripatti S., Pirinen M. (2017). Prospects of Fine-Mapping Trait-Associated Genomic Regions by Using Summary Statistics from Genome-wide Association Studies. Am. J. Hum. Genet..

[45] Pirinen M. (2023). linemodels: clustering effects based on linear relationships. Bioinformatics.

[46] Stan Development Team (2019). Stan Modeling Language Users Guide and Reference Manual 2.21.0.

[47] Flynn E., Tanigawa Y., Rodriguez F., Altman R.B., Sinnott-Armstrong N., Rivas M.A. (2021). Sex-specific genetic effects across biomarkers. Eur. J. Hum. Genet..

[48] Berisa T., Pickrell J.K. (2016). Approximately independent linkage disequilibrium blocks in human populations. Bioinformatics.

[49] Rawlik K., Canela-Xandri O., Tenesa A. (2016). Evidence for sex-specific genetic architectures across a spectrum of human complex traits. Genome Biol..

[50] Ge T., Chen C.-Y., Neale B.M., Sabuncu M.R., Smoller J.W. (2017). Phenome-wide heritability analysis of the UK Biobank. PLoS Genet..

[51] Bernabeu E., Canela-Xandri O., Rawlik K., Talenti A., Prendergast J., Tenesa A. (2021). Sex differences in genetic architecture in the UK Biobank. Nat. Genet..

[52] Yang M.-L., Xu C., Gupte T., Hoffmann T.J., Iribarren C., Zhou X., Ganesh S.K. (2024). Sex-specific genetic architecture of blood pressure. Nat. Med..

[53] Connelly P.J., Casey H., Montezano A.C., Touyz R.M., Delles C. (2022). Sex steroids receptors, hypertension, and vascular ageing. J. Hum. Hypertens..

[54] Ohlsson C., Wallaschofski H., Lunetta K.L., Stolk L., Perry J.R.B., Koster A., Petersen A.-K., Eriksson J., Lehtimäki T., Huhtaniemi I.T. (2011). Genetic Determinants of Serum Testosterone Concentrations in Men. PLoS Genet..

[55] Heid I.M., Jackson A.U., Randall J.C., Winkler T.W., Qi L., Steinthorsdottir V., Thorleifsson G., Zillikens M.C., Speliotes E.K., Mägi R. (2010). Meta-analysis identifies 13 new loci associated with waist-hip ratio and reveals sexual dimorphism in the genetic basis of fat distribution. Nat. Genet..

[56] Pazokitoroudi A., Chiu A.M., Burch K.S., Pasaniuc B., Sankararaman S. (2021). Quantifying the contribution of dominance deviation effects to complex trait variation in biobank-scale data. Am. J. Hum. Genet..

[57] Zito A., Davies M.N., Tsai P.-C., Roberts S., Andres-Ejarque R., Nardone S., Bell J.T., Wong C.C.Y., Small K.S. (2019). Heritability of skewed X-inactivation in female twins is tissue-specific and associated with age. Nat. Commun..

[58] Brix T.H., Knudsen G.P.S., Kristiansen M., Kyvik K.O., Ørstavik K.H., Hegedüs L. (2005). High Frequency of Skewed X-Chromosome Inactivation in Females with Autoimmune Thyroid Disease: A Possible Explanation for the Female Predisposition to Thyroid Autoimmunity. J. Clin. Endocrinol. Metab..

[59] Pirastu N., Cordioli M., Nandakumar P., Mignogna G., Abdellaoui A., Hollis B., Kanai M., Rajagopal V.M., Parolo P.D.B., Baya N. (2021). Genetic analyses identify widespread sex-differential participation bias. Nat. Genet..

[60] McAllister K., Mechanic L.E., Amos C., Aschard H., Blair I.A., Chatterjee N., Conti D., Gauderman W.J., Hsu L., Hutter C.M. (2017). Current Challenges and New Opportunities for Gene-Environment Interaction Studies of Complex Diseases. Am. J. Epidemiol..

[61] Motsinger-Reif A.A., Reif D.M., Akhtari F.S., House J.S., Campbell C.R., Messier K.P., Fargo D.C., Bowen T.A., Nadadur S.S., Schmitt C.P. (2024). Gene-environment interactions within a precision environmental health framework. Cell Genom..

