# Supplementary material for: Role of X chromosome and dosage-compensation mechanisms in complex trait genetics
Source: Am J Hum Genet. 2025 May 12;112(6):1330–43. doi: 10.1016/j.ajhg.2025.04.004 (PMC12256796; doi:10.1016/j.ajhg.2025.04.004)
Supplement: Document S1. Figures S1–S20, Tables S1, S6, S8, S9, S11, and S12, supplemental notes, and supplemental acknowledgments [file mmc1.pdf]

The American Journal of Human Genetics, Volume *112*

Supplemental information

Role of X chromosome and dosage-compensation mechanisms in complex trait genetics

Yu Fu, Aino Kenttämies, Sanni Ruotsalainen, Matti Pirinen, and Taru Tukiainen

Supplemental Notes

Motivation and consequence of alternative coding system in chrX

ChrX can also be coded as the count of observed alleles in males and females[1]. Assuming full XCI, a half of the active allele effect is being estimated in females and the full active allele effect in males. Thus, the observed male effects are expected to be twice the female effects when the active allele effect sizes are equal between the sexes (Table S1). In females, $\beta_X = \beta_A$ corresponds to two-fold larger a_X than a_A, while in males, $\beta_X = \beta_A$ corresponds to equal a_X and a_A. On the other hand, if there was no XCI and both alleles in females were fully functional, then this approach would be estimating the full active allele effect also in females. Such an approach has been used for studying escape from XCI [2–4]. However, in general, this may be an unrealistic assumption as genes escaping from XCI were rarely fully expressed[5].

A biologically intuitive approach is to count each allele in females as 0.5 copies and in males as one copy as implemented in SNPTEST v2.5[6]. This method automatically takes into account full XCI and is estimating the active allele effect in both sexes (Table S1). Thus, when comparing with autosomal effect sizes, a similar magnitude of chrX effect would suggest an equal a_X and a_A.

FinnGen ethics statement

All participants in FinnGen provided informed consent for biobank research, based on the Finnish Biobank Act. Cohorts collected before the start of FinnGen (August 2017) were collected under study-specific consents and subsequently transferred to the Finnish biobanks following approval from Valvira, the National Supervisory Authority for Welfare and Health. Recruitment protocols followed the biobank

protocols approved by Valvira. The Coordinating Ethics Committee of the Hospital District of Helsinki and Uusimaa approved the FinnGen study protocol Nr HUS/990/2017. The FinnGen study is approved by Finnish Institute for Health and Welfare (THL), approval number THL/2031/6.02.00/2017, amendments THL/1101/5.05.00/2017, THL/341/6.02.00/2018, THL/2222/6.02.00/2018, THL/283/6.02.00/2019, THL/1721/5.05.00/2019, Digital and population data service agency VRK43431/2017-3, VRK/6909/2018-3, VRK/4415/2019-3 the Social Insurance Institution (KELA) KELA 58/522/2017, KELA 131/522/2018, KELA 70/522/2019, KELA 98/522/2019, and Statistics Finland TK-53-1041-17. The Biobank Access Decisions for FinnGen samples and data utilized in FinnGen Data release 10 include: THL Biobank BB2017_55, BB2017_111, BB2018_19, BB_2018_34, BB_2018_67, BB2018_71, BB2019_7, BB2019_8, BB2019_26, BB2020_1, BB2021_65, Finnish Red Cross Blood Service Biobank 7.12.2017, Helsinki Biobank HUS/359/2017, HUS/248/2020, HUS/150/2022 §12, §13, §14, §15, §16, §17, §18, and §23, Auria Biobank AB17-5154 and amendment #1 (August 17 2020) and amendments BB_2021-0140, BB_2021-0156 (August 26 2021, Feb 2 2022), BB_2021-0169, BB_2021-0179, BB_2021-0161, AB20-5926 and amendment #1 (April 23 2020)and its modification (Sep 22 2021), Biobank Borealis of Northern Finland_2017_1013, 2021_5010, 2021_5018, 2021_5015, 2021_5023, 2021_5017, 2022_6001, Biobank of Eastern Finland 1186/2018 and amendment 22§/2020, 53§/2021, 13§/2022, 14§/2022, 15§/2022, Finnish Clinical Biobank Tampere MH0004 and amendments (21.02.2020 & 06.10.2020), §8/2021, §9/2022, §10/2022, §12/2022, §20/2022, §21/2022, §22/2022, §23/2022, Central Finland Biobank 1-2017, and Terveystalo Biobank STB 2018001 and amendment 25th Aug 2020,

Finnish Hematological Registry and Clinical Biobank decision 18th June 2021, Arctic biobank P0844: ARC_2021_1001.

Validation of GENESIS

We validated the performance of GENESIS by comparing the estimated h_A^2 with the corresponding estimate from LDSC[7], a tool applicable to autosomal data only. Sex-specific estimates from the two methods were well aligned (Pearson r = 0.8 in both sexes) with most traits (36/48 in females and 34/48 in males) showing no detectable differences between the methods (*P*-value ≥ 0.05, two samples *Z* test; Figure S14, Table S3).

Number of genome-wide significant variants

We observed a clear sex difference in the numbers of significantly associated X-linked loci. At the genome-wide significance threshold of 5×10^{-8}, we detected a 2.3-fold (SE = 0.19) the number of associated LD independent variants in males (range from 0 to 25 per trait, median 3) compared to females (range from 0 to 13 per trait, median 1) (Figure S3A, Table S6). These observations did not seem to be driven by sex-specific genetic architecture in chrX as male and female effect estimates of these variants identified from sex-specific analyses were similarly highly correlated in chrX as in the autosomes (Figure S15). The differences in the number of detected associations can rather be explained by greater statistical power in males than in females to detect genetic associations in chrX, a difference arising from the unique biology of chrX. In comparison, in the autosomes, we observed slightly more independently associated genome-wide significant loci in females than in males (1.2-fold (SE = 0.03), Figure S3B), a finding at least partly attributable to the

larger sample size of the female subsets for all traits except for direct bilirubin and testosterone (on average 7.62% more females than males; Table S2).

The effect of allele frequency and functional consequences on active allele effects

As variants with low frequency and severe functional consequences tend to have large effects, we assessed if the differences in the effect size estimates were explained by differences in minor allele frequencies (MAF) and functional consequences between autosomes and chrX. We first examined how chrX differs from autosomes in general using ~1.1 million independent common variants from the GENESIS 1000 Genomes European reference panel. We observed only minor differences in the allele frequency distribution between chrX and autosomes — variants in chrX have overall a slightly higher MAF (median 0.253 versus 0.251; *P*-value < 0.001, *t*-test), and also a slightly larger proportion of less common variants (0.01 < MAF < 0.05) (1.7% versus 1.4%; *P*-value < 5×10^{-6}, χ^2 test; Figures S8A and S8B) relative to autosomes (here we note that more than 75% of low frequency variants were with MAF ≥ 0.04). Further testing the relationship of MAF and a at the identified sex-specific trait-associated variants, we observed significantly larger a_X compared to a_A across all the MAF bins (Figures S8C and S8D), suggesting limited effect of MAF on the difference of a between chrX and autosomes.

With regard to variants' functional consequences, we observed differences in the active allele effect distributions between chrX and the autosomes. In general, the common variants in chrX were depleted in regulatory and coding regions (predicted by Variant Effect Predictor[8]; Figure S9) and showed enrichment for less pathogenic consequences (estimated by the CADD score[9]; Figure S10A) relative to autosomes.

This reduced density of functional variants in chrX aligns with the stronger selection pressure on chrX arising from the hemizygosity of chrX in males[10,11]. Across the trait-associated variants, we again observed larger a_X compared to a_A but this difference was driven by variants with regulatory and other less severe functional impacts whereas no difference between a_X and a_A was observed at the coding region variants (6 (10%) and 226 (4.3%) of the chrX and autosomal lead variants, respectively) (Figure S9B). The same phenomenon was observed when grouping variants based on their pathogenicity. For female lead variants with the greatest pathogenicity (3 (5.0%) and 139 (2.6%) in chrX and autosomes, respectively), we observed no difference between a_X and a_A (median 0.056 versus 0.051, *P*-value = 0.48, Wilcoxon rank-sum test) (Figure S10B); however, for variants predicted as less pathogenic (scaled CADD score ≤ 20), we observed a significantly larger a_X compared to a_A (median 0.046 versus 0.026, *P*-value = 2.12×10^{-22}, Wilcoxon rank-sum test).

Across all comparisons, the observed patterns were not affected by pleiotropy (Figures S8E, S8F, S9C and S10C).

Dosage compensation ratio analysis

Following the work by [2], we estimated chrX and autosomal DC ratio (DCR) using directly the $\widehat{h^2_{X,m}}$ and $\widehat{h^2_{X,f}}$, $\widehat{h^2_{a,m}}$ and $\widehat{h^2_{a,f}}$ estimated with GENESIS for 34 traits with non-zero h^2_X in both sexes. The DCR and its corresponding standard error were estimated as[2]:

$$DCR = \frac{\widehat{h^2_m}}{\widehat{h^2_f}}$$

$$SE(DCR) = \frac{\widehat{h_m^2}}{\widehat{h_f^2}} \sqrt{\left(\frac{SE^2\left(\widehat{h_m^2}\right)}{\widehat{h_m^2}^2} + \frac{SE^2\left(\widehat{h_f^2}\right)}{\widehat{h_f^2}^2}\right)}$$

where $\widehat{h_m^2}$ and $\widehat{h_f^2}$ are h^2 estimates from GENESIS in males and females and $SE\left(\widehat{h_m^2}\right)$ and $SE\left(\widehat{h_f^2}\right)$ are corresponding standard errors. ChrX DCRs (DCR_X) were estimated with chrX estimates $\widehat{h_{X,m}^2}$ and $\widehat{h_{X,f}^2}$ with corresponding standard errors and autosomal DCRs (DCR_A) with autosomal estimates $\widehat{h_{A,m}^2}$ and $\widehat{h_{A,f}^2}$ with standard errors.

We compared our estimated DCR with DCR estimated using summary statistics as[2]. Our DCR estimates were correlated with that estimated with summary statistics (Pearson r = 0.93 and 0.40 for autosomes and chrX, respectively; Figure S16). DCR estimated with summary statistics had much smaller standard errors and we observed discordant DCR estimates for urate, sex-hormone binding globin, and waist-to-hip ratio in autosomes and testosterone in chrX, which may be due to the DCR estimated with summary statistics being less robust to regional sex difference given the lack of consideration of LD.

As sex difference in h^2 were observed for autosomes (first section of results), we adjusted the chrX DCR estimates with the corresponding autosomal DCRs to account for sources of sex differences in h^2 that are unrelated to chrX specific biology, assuming the effect of e.g. environmental sex biases reflected similarly in autosomes and chrX h^2 (Table S3):

$$DCR_{X,adjusted} = \frac{DCR_X}{DCR_A}$$

$$SE\left(DCR_{X,adjusted}\right) = \frac{DCR_X}{DCR_A} \sqrt{\left(\frac{SE^2(DCR_X)}{{DCR_X}^2} + \frac{SE^2(DCR_A)}{{DCR_A}^2}\right)}$$

where DCR_X and DCR_A are estimated DCR in chrX and autosomes, respectively and $SE(DCR_X)$ and $SE(DCR_A)$ are corresponding standard errors in chrX and autosomes, respectively.

For most traits, the adjustment did not introduce overwhelming changes (mean unadjusted 2.46 versus adjusted 2.42; Figure S17) as most autosomal DCR estimates were close to one. The most pronounced change was observed for testosterone (unadjusted 4.67 (SE = 1.92) versus adjusted 2.86 (SE = 1.24)).

We used DCR to test the three XCI scenarios – full XCI (F-XCI), escape XCI (E-XCI) and no XCI (N-XCI). Across the traits, we observed a mean adjusted DCR of 2.40 (SD = 1.15) suggesting, in general, concordance with F-XCI and E-XCI rather than N-XCI, as expected given the existing evidence for XCI[2]. At the individual trait level, while we observed the adjusted DCRs of diastolic (0.71 (SE = 0.35) and systolic blood pressures (0.61 (SE = 0.23)) aligned with the expected value under N-XCI (DCR=0.5), echoing our previous results, the DCR metric did not distinguish between F-XCI (DCR=2) and E-XCI (DCR=1.75) for any of the traits (Figure S18). We observed DCRs greater than 2 for albumin, creatinine, and body fat and mass related traits (body fat mass, weight, impedance of body and left leg), of which correlated traits, body fat percentage and basal metabolic rate, have been reported with DCR greater than 2 due to substantial sex difference in SNP effects in two regions near the *FAM9A/FAM9B* genes and near the *AR* gene[2], that are thought to reflect fat-reducing effects of androgen in males. We additionally calculated DCRs using FinnGen data for height, BMI and weight. While we observed similar DCRs for height as in the UKB, the DCR estimates for weight and BMI differed (Figure S18; Tables S3).

Sex-biased effect analysis

Four-component sex bias mixture model

Demonstrating the consistency of our model, we observed the point estimates of the null effect proportion negatively correlated with h^2 estimates in both chrX (Spearman r = -0.40 in females and -0.30 in males) and autosomes (Spearman r = -0.60 in females and -0.63 in males) across traits with nonzero h_X^2 in both sexes (Figure S19).

Three different prior distributions of σ^2 were tested: Inverse-Gamma(1,1), Inverse-Gamma(0.001,0.001) and Uniform(0,1). We compared the estimated parameters with these priors using summary statistics of height for chrX variants. We calculated the expected log-predictive density with leave-one-out cross-validation (ELPD-LOO) for each prior with "loo" R package and compared ELPD-LOO across different priors (Figure S20). The comparison indicated differences between the priors for σ^2, and ELPD-LOO was the highest for Uniform(0,1) prior. Therefore, we chose to use Uniform(0,1) as the prior for σ^2 in our analyses.

Male-biased effects in chrX in testosterone genetics

Testosterone has been shown in previous research[12–14] to exhibit sex-specific genetic architecture in the autosomes. We observed, as expected, systematically larger effects in males across the genome, however, compared to autosomes, chrX was more enriched with male-biased variants (scaled proportion 95.3% (95% CI: 85.5 – 99.7%) versus 78.7% (95% CI: 55.9 – 94.6%); Table S10)), an observation consistent with a previous study focusing on sex-specific effects[12]. Such pattern supports the predicted enrichment of variants in chrX that affect traits towards the male optimum[15]. For example, among the 12 lead variants for testosterone in chrX that show male-biased effects, six loci (rs12015400, X:65779624_GTT_G,

rs189261721, rs146415516, rs140812443, rs7052964) are close to genes involved in the androgen receptor pathway (*AR* (androgen receptor), *EDA2R* (ectodysplasin A2 Receptor), *KLF8* (KLF transcription factor 8)[16]) and one (rs112265145) close to *FAM9A/FAM9B* region related to spermatogenesis in adults[17] (Table S13). Three regions associated with testosterone showed pleiotropic male-specific effects (Table S13), two of which have been identified as sex-heterogeneous regions[2]: in the *FAM9A/FAM9B* region led by rs112265145 in testosterone association, loci associated with impedance of body, phosphate, heel bone mineral density, insulin-like growth factor 1, total bilirubin, and creatinine display male-specific effects; within the well-known androgen associated locus[2,18,19], *EDA2R*/*AR* region in Xq12, we observed loci associated with apolipoprotein A, apolipoprotein B, high-density lipoprotein, waist-to-hip ratio, vitamin D, body fat mass, triglyceride, and creatinine all show considerable larger effects in males except for apolipoprotein B associated rs35176586 showing slightly larger effects in females; in the *RTL9*/*CHRDL1* region led by rs881090 in testosterone association in Xq23, a known lipid-associated region[20], we observed lead variants associated apolipoprotein B, cholesterol, low-density lipoprotein, albumin, sex hormone-binding globulin, aspartate aminotransferase and calcium all display male-biased effects except for X:109833687_GGT_G association with calcium shows a female-biased effect.

Lack of female-biased effects in chrX in waist-to-hip ratio genetics

Out of 8 lead variants associated with waist-to-hip ratio in chrX, we identified only two with female-biased effects – rs113303918 in the intron of *FHL1* (four and a half LIM domains 1) and rs35318931, a missense variant in *SRPX* (sushi repeat containing protein X-Linked) (Table S13), consistent with previous findings in UKB[2].

rs4419961 in the *EDA2R* /*AR* region was identified having larger effects in males on waist-to-hip ratio (Table S13).

Replicability of sex-biased effects

To understand the poor replicability of the female-biased variants across biobanks, we asked if the sex-specific effects differed between the biobanks. For the sex-combined lead variants for height, we observed strong correlations between UKB and FinnGen in both female (chrX: Pearson r = 0.88, sign test for sign concordance *P*-value = 3.18×10^{-13}; autosomes: Pearson r = 0.93, sign test *P*-value < 10^{-15}) (Figures S12C and 13C) and male effects (chrX: Pearson r = 0.92, sign test *P*-value = 3.18×10^{-13}; autosomes: Pearson r = 0.93, sign test *P*-value < 10^{-15}) (Figures S12D and S13D), confirming the genetic effects on height in both autosomes and chrX are highly reproducible across data sets. Following these observations, we asked if the sex differences in effect estimates, measured as the sex difference z-scores, correlate between the biobanks. We found these z-scores weakly correlated between the two biobanks, with a small enrichment in directionally concordant effects in the autosomes (chrX: Pearson r = 0.32, sign test for sign concordance *P*-value = 0.78; autosomes: Pearson r = 0.13, sign test *P*-value = 0.004) (Figures S12B and S13B). Overall, we found little evidence of consistency in sex differences in the effect sizes for human height between the two biobanks.

Supplemental Figures

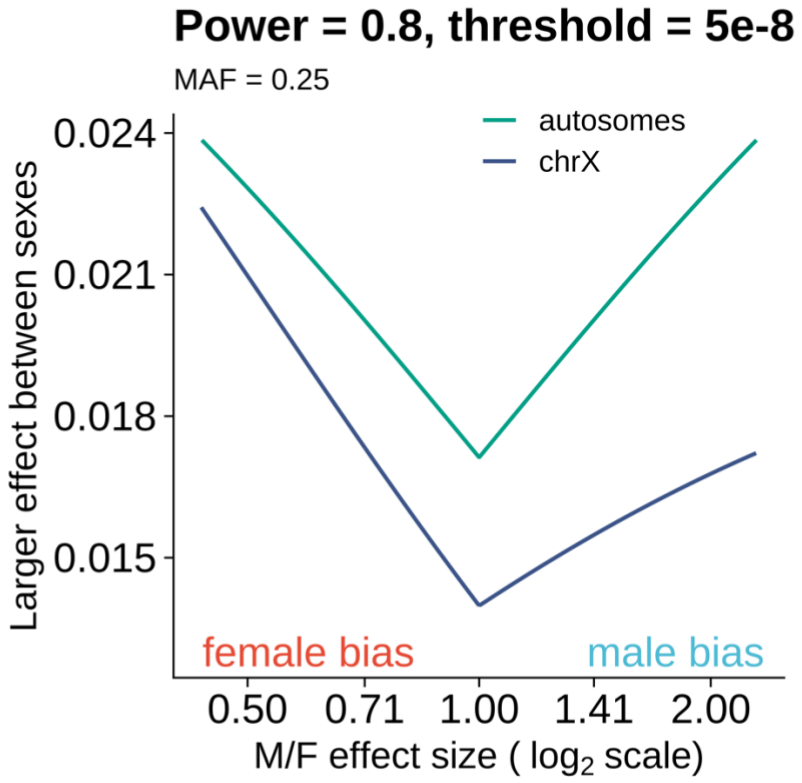

Figure S1. Illustration of the power bias in a sex-combined genome-wide association study (GWAS) in chrX.

To exemplify the power bias in chrX, we calculated the magnitude of the male or female effect size needed for 80% power to detect a SNP with MAF = 0.25 in chrX or in an autosome in a sex-combined GWAS with equal sample sizes of males and females ($n_m = n_f = 180{,}000$) at genome-wide significance level of 5×10^{-8}. Under full XCI, where male-to-female effect size ratio is one, we were able to detect SNPs with effect size of ~0.014 in chrX while a larger effect size of ~0.017 was required in autosomes. If effect sizes differed between the sexes, we would be able to detect the sex-biased SNPs symmetrically in autosomes but with a bias favoring the male-biased SNPs in chrX in a sex-combined analysis. For instance, in a sex-combined chrX analysis, with a power of 80%, we can only detect a female-biased variants with

twofold larger effects in females if the underlying female effect is as large as 0.021, while for a male-biased variants with twofold larger effects in males the underlying male effects need only be 0.017.

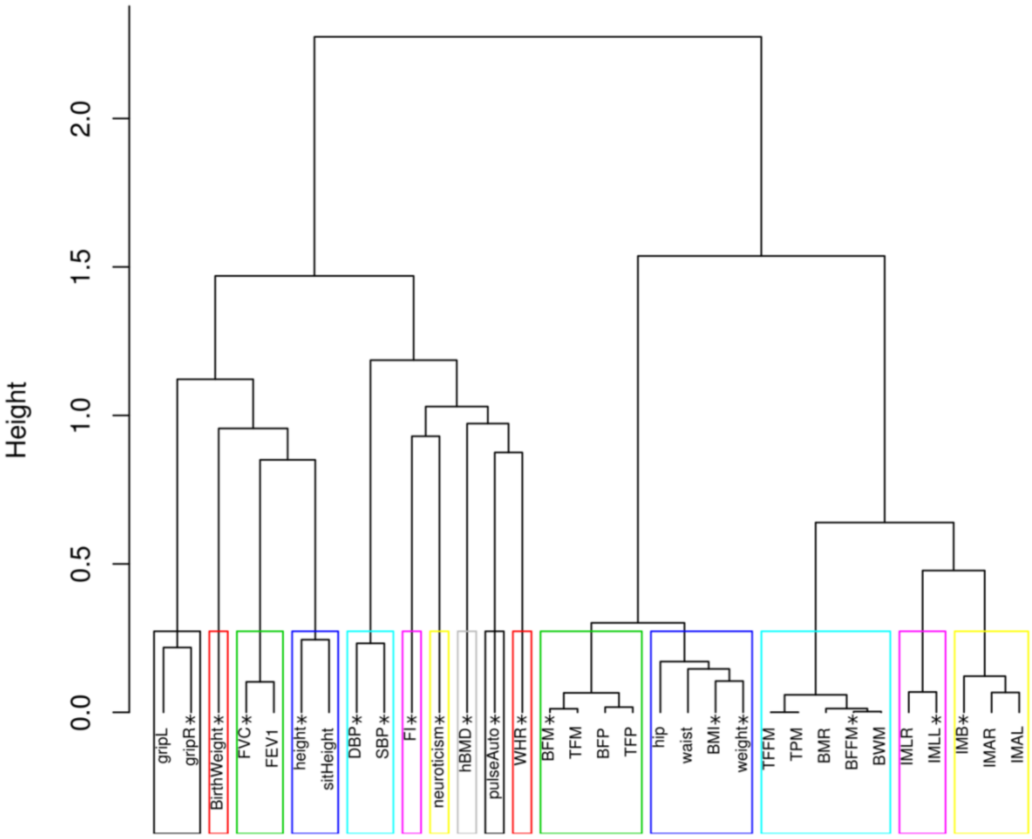

Figure S2. Hierarchical clustering dendrogram and identified clusters based on the correlation of adjusted and normalized trait values in the sex-combined population.

The traits included in this study for GWAS are denoted with asterisks. Traits: hand grip strength, left (gripL) and right (gripR), birth weight (BirthWeight), forced vital capacity (FVC), forced expiratory volume in 1-second (FEV-1), standing height (height), sitting height (sitHeight), diastolic blood pressure (DBP), systolic blood pressure (SBP), fluid intelligence score (FI), neuroticism score (neuroticism), heel bone mineral density T-score (hBMD), automated reading pulse rate (pulseAuto),

waist-to-hip ratio (WHR), whole body fat mass (BFM), trunk fat mass (TFM), body fat percentage (BFP), trunk fat percentage (TFP), hip circumference (hip), waist circumference (waist), body mass index (BMI), trunk fat-free mass (TFFM), trunk predicted mass (TPM), basal metabolic rate (BMR), whole body fat-free mass (BFFM), whole body water mass (BWM), impedance of leg, right (IMLR) and left (IMLL), impedance of whole body (IMB), impedance of arm, right (IMAR) and left (IMAL).

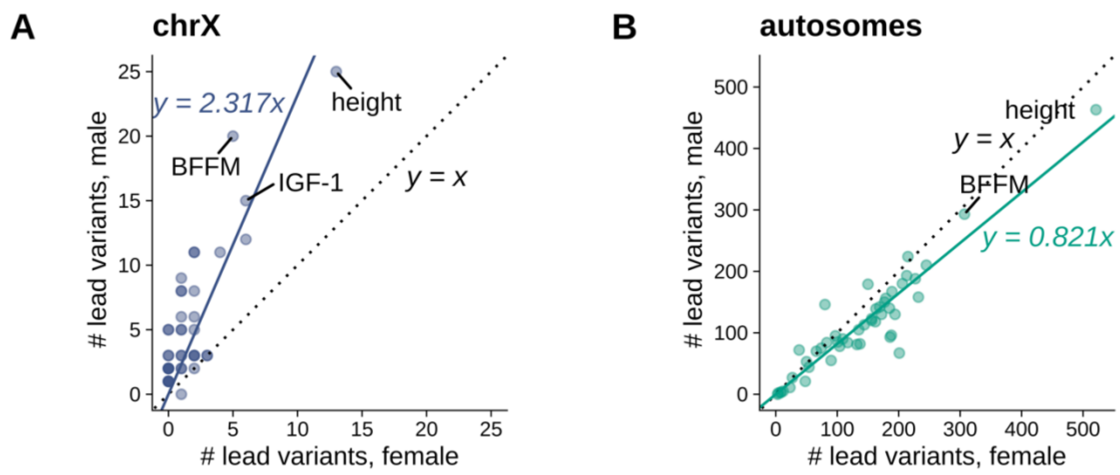

Figure S3. Comparison of the number of lead variants in male and female GWAS across 48 traits.

(A) The comparison in chrX and (B) the comparison in autosomes. The dotted line indicates equal number of lead variants in male and female GWAS. The solid lines are regression lines. The numerical values are reported in Table S6. Abbreviations: whole body fat-free mass (BFFM), insulin-like growth factor 1 (IGF-1).

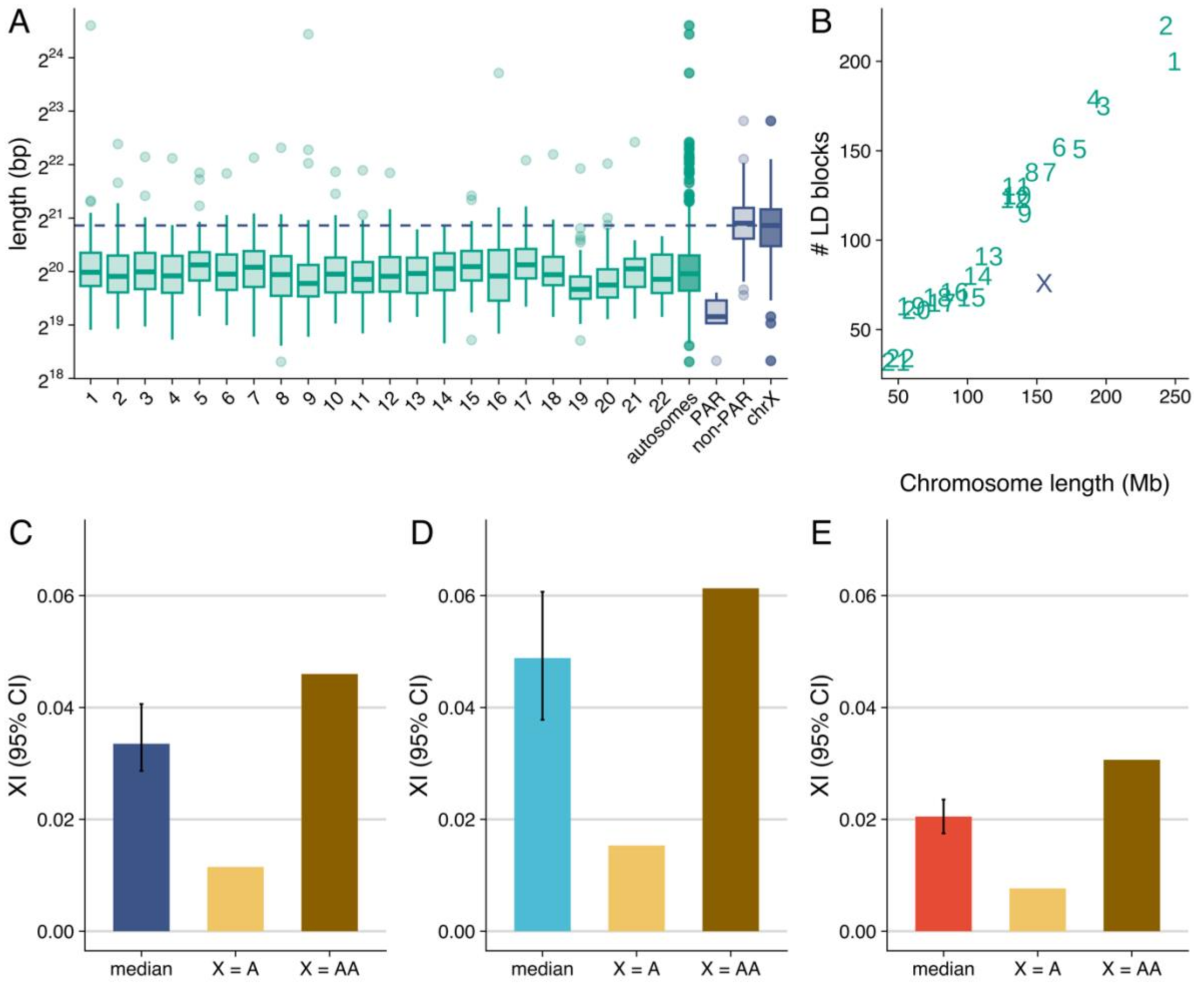

Figure S4. Comparison of chrX and autosomes based on the number of LD blocks.

(A) Distribution of lengths of linkage disequilibrium (LD) blocks for each autosome, all autosomes, PAR and non-PAR of chrX and all chrX, shown as boxplots. The dashed line indicates the chrX median. The median LD block length in chrX is approximately twice that of the median autosome LD block length reflecting the reduced recombination in chrX. (B) The number of LD blocks in each chromosome versus the total length of the chromosome. Numerical values are reported in Table S8. Owing to the more extensive LD, the number of LD blocks in chrX is less than in autosomal chromosomes of similar length. (C-E) The median XI (bootstrap 95% CI) contrasted to the expected XI based on the number of LD blocks when the genetic

effect of one active chrX is equal to one autosome (X = A) or equal to a pair of autosomes (X = AA) (C) in the overall population, (D) in males, and (E) in females for 35 traits with nonzero h_X^2 in both sexes. Numerical values are reported in Table S3.

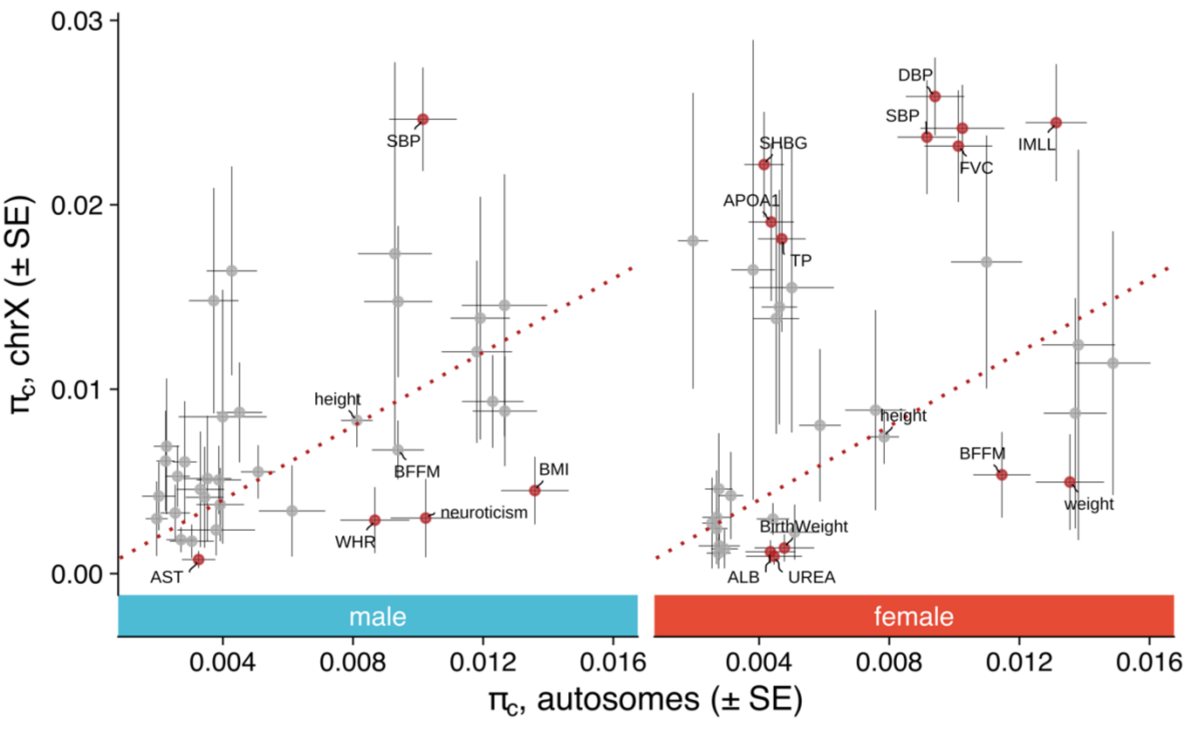

Figure S5. Comparison of the estimated proportion of causal variants (π_c) in chrX and in autosomes across 35 traits with nonzero h_X^2 in both sexes. Results are shown separately in males and in females. The dotted line indicates equal proportion of causal variants in chrX and autosomes. Numerical values reported in Tables S4 and S5. Abbreviations: systolic blood pressure (SBP), diastolic blood pressure (DBP), whole body fat-free mass (BFFM), body mass index (BMI), waist-to-hip ratio (WHR), aspartate aminotransferase (AST), impedance of leg, left (IMLL), forced vital capacity (FVC), sex hormone-binding globulin (SHBG), apolipoprotein A (APOA1), total protein (TP), albumin (ALB), urea (UREA).

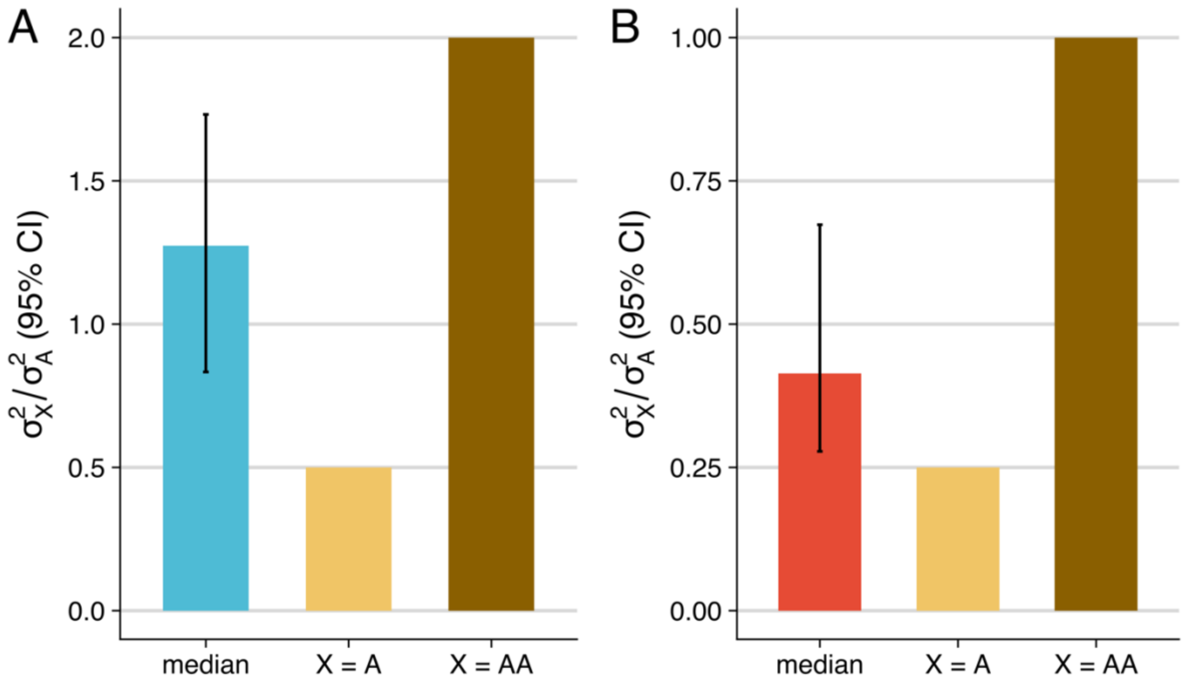

Figure S6. Comparison of σ^2 in chrX and in autosomes.

(A) The comparison in males and (B) the comparison in females. The blue and red bars indicate the median of σ_X^2/σ_A^2 over the traits in males and females, respectively. The light and dark brown bars indicate the expected relationship between autosomal and chrX σ^2 under X = AA and X = A. Numerical values reported in Tables S4 and S5.

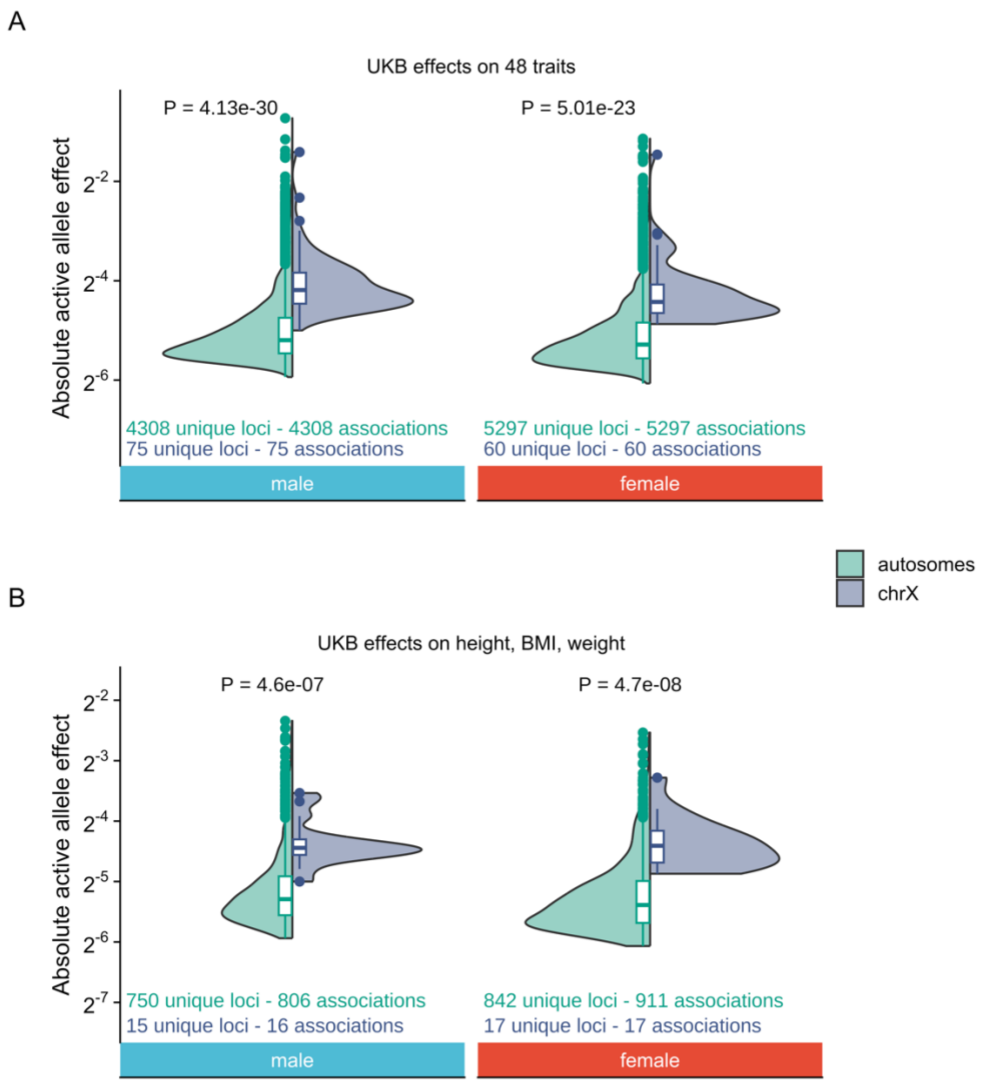

Figure S7. Comparison of a_X and a_A for trait-associated variants.

(A) Comparison in sex-specific unique trait-associated variants, where one trait was randomly selected for pleiotropic loci. (B) Comparison in variants associated with height, BMI, and weight in UKB with a estimated in UKB (for comparison with Figure 3B). Male GWAS in non-PAR have been down-sampled by half to achieve similar statistical power as in GWAS in autosomes. Numerical values are reported in Table S7 for UKB estimates and FinnGen summary statistics for FinnGen estimates.

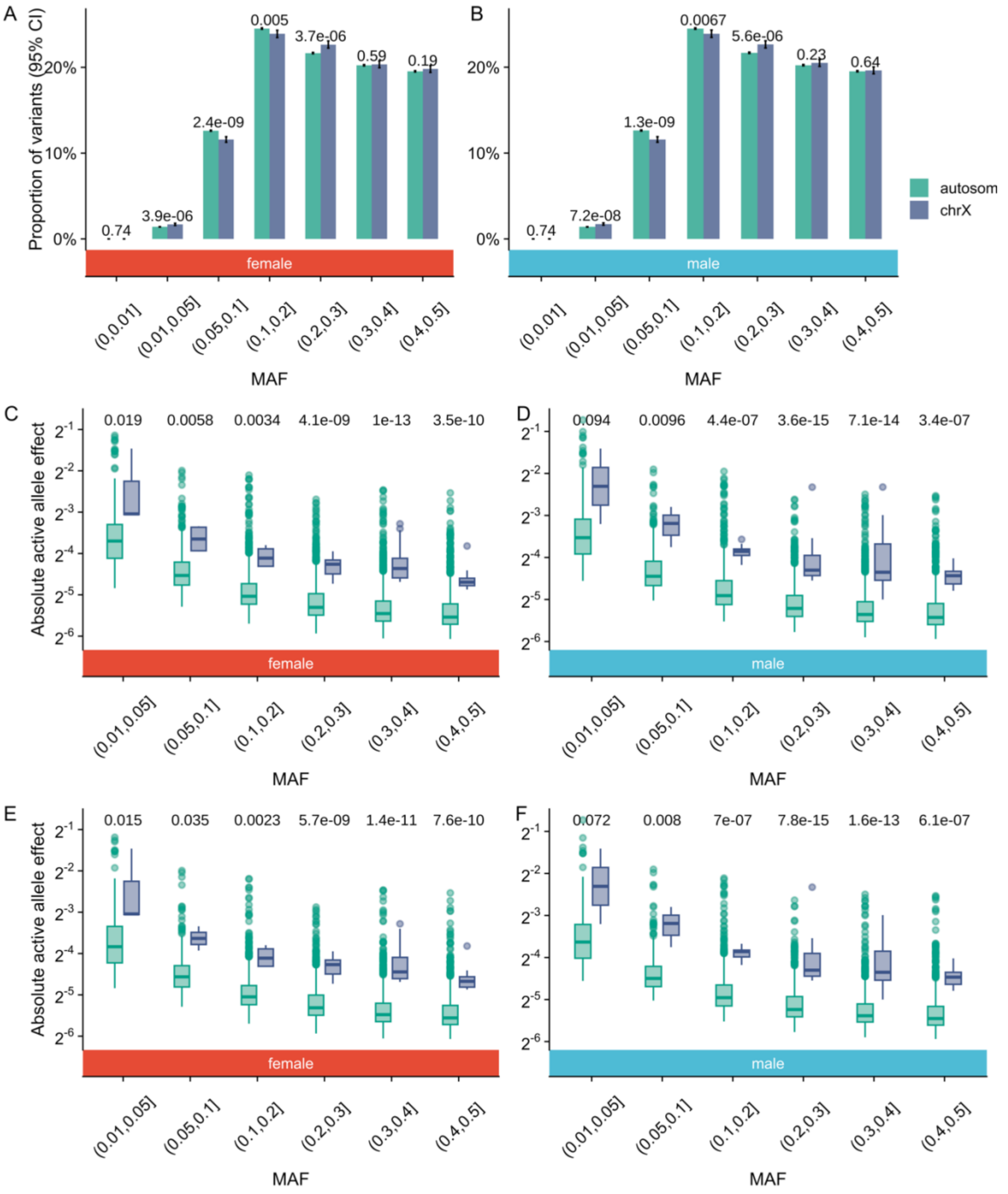

Figure S8. Comparison of MAF and the effects of MAF on a between autosomes and chrX.

We compared the MAF distributions of variants included in the GENESIS reference panel between autosomes and chrX, with MAF calculated in (A) female and (B) male UKB samples. *P*-values of proportion difference between autosomes and chrX (χ^2 test) are indicated on top of each pair of bars. The distribution of a of (C) female and

(D) male lead variants for each MAF bin in autosomes and chrX across all associations. The male GWAS in non-PAR has been down-sampled by half. The distribution of a of (E) female and (F) male lead variants for each MAF bin in autosomes and chrX, where one trait was randomly selected for pleiotropic variants. *P*-values of difference between a_X and a_A (Wilcox rank-sum test) were indicated on the top of the figures. Numerical values are reported in Table S7 for lead variants.

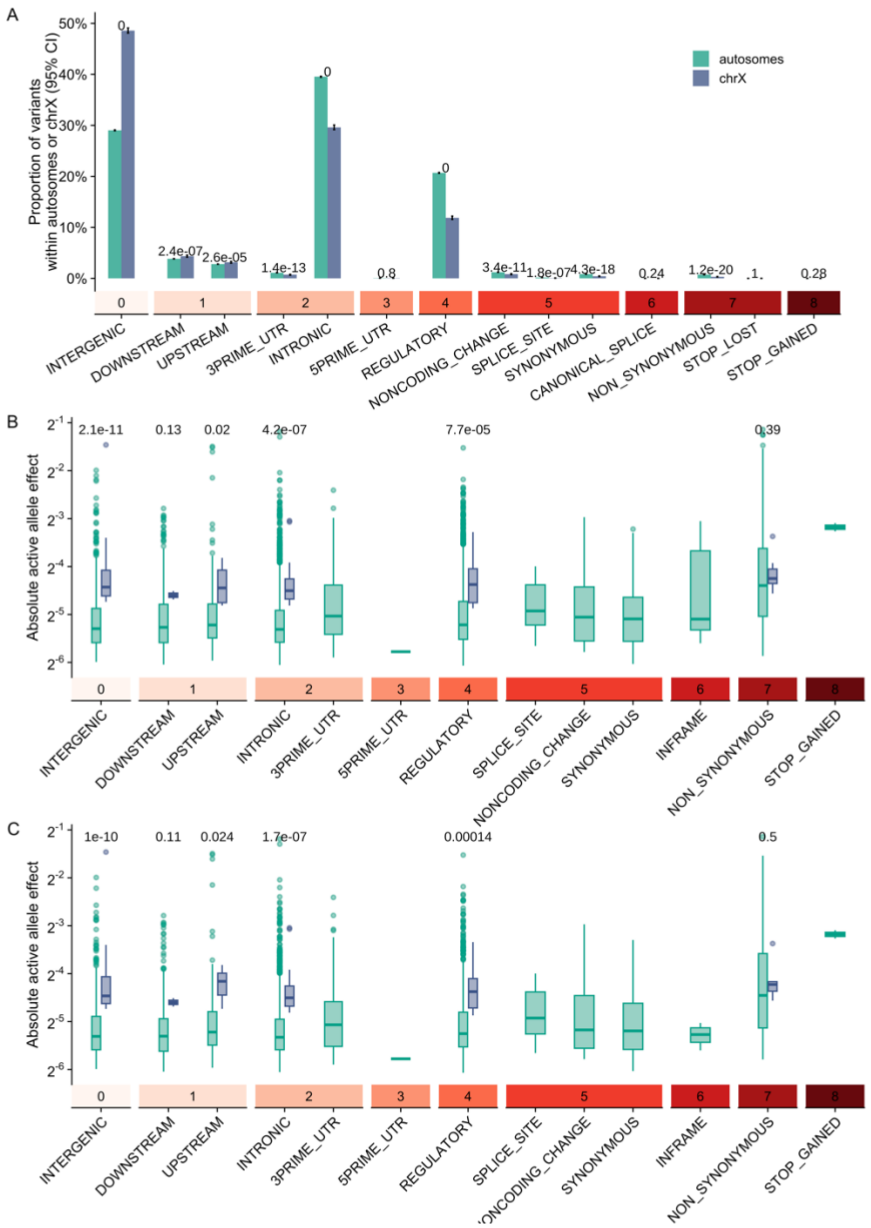

Figure S9. Comparison of functional consequences and the effects of functional consequences on active allele effect a between autosomes and chrX.

We compared (A) the proportions of functional consequences between common variants used in the GENESIS reference panel between autosomes and chrX. *P*-values of proportion difference between autosomes and chrX (χ^2 test) are indicated on top of each pair of bars. We compared the a_X and a_A of female lead variants (B) across all associations and (C) with a single effect randomly selected for pleiotropic

variants in the same functional consequence. *P*-values of difference between a_X and a_A (Wilcox rank-sum test) were indicated on the top of the figures. Numerical values are reported in Table S7 for lead variants. The numerical values and color of the blocks on top of the functional consequences indicate the severity of the consequences.

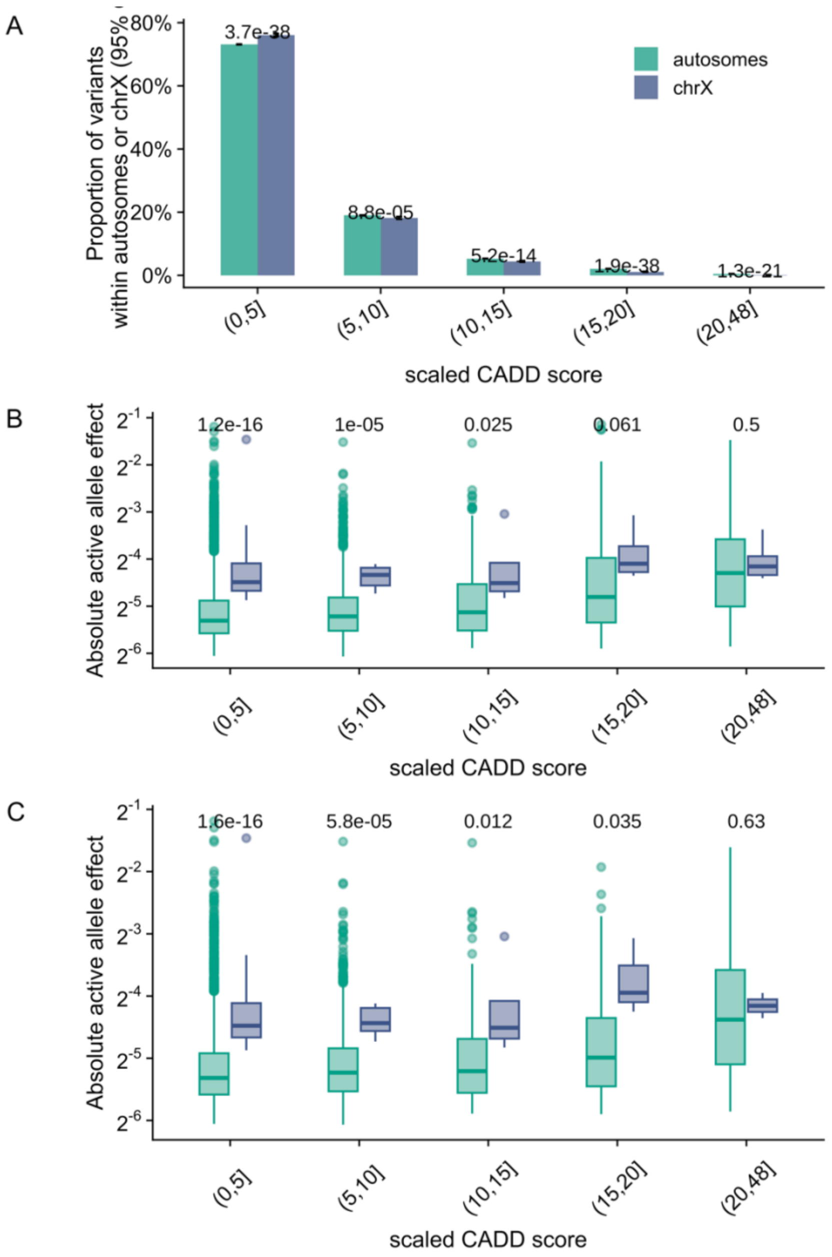

Figure S10. Comparison of pathogenicity measured as scaled CADD score and the effect of pathogenicity on a between autosomes and chrX.

The higher the scaled CADD score, the more pathogenic the variant is predicted to be. We compared (A) the proportions of scaled CADD scores between variants included in the GENESIS reference panel in autosomes and in chrX. *P*-values of proportion difference between autosomes and chrX (χ^2 test) were indicated on top of each pair of bars. We compared (B) the a_X and a_A of female lead variants within in

the same CADD bin and (C) with a single a randomly selected for pleiotropic variants within each bin. *P*-values of difference between a_X and a_A (Wilcox rank-sum test) were indicated on the top of the figures. Numerical values are reported in Table S7 for lead variants.

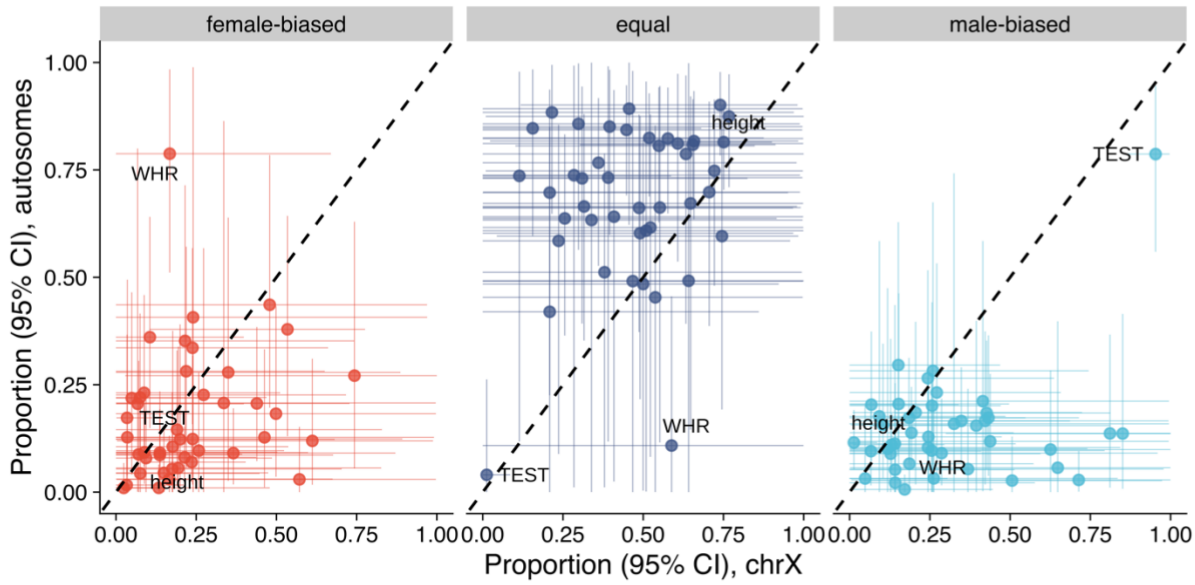

Figure S11. Comparison of the estimated non-null proportions of female-biased, equal, and male-biased components between chrX and autosomes. Numerical values are reported in Table S10. Abbreviations: waist-to-hip ratio (WHR), testosterone (TEST).

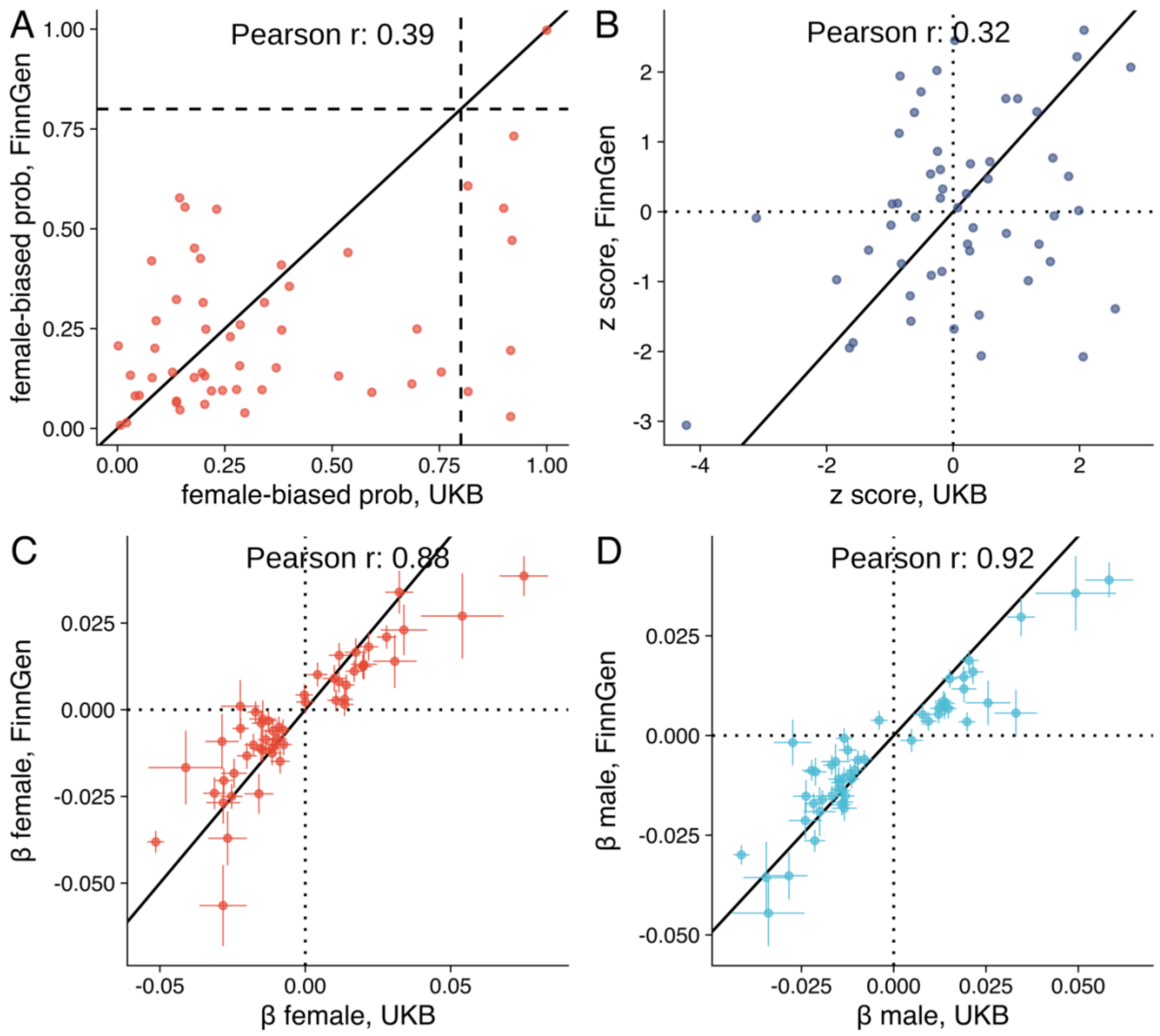

Figure S12. Comparison of UKB and FinnGen results.

The comparison in (A) female-biased probability, (B) sex difference z-score, (C) female effect sizes (± SE) and (D) male effect sizes (± SE) of lead variants identified in UKB height sex-combined GWAS in chrX. Numerical values are reported in Tables S13 and S14.

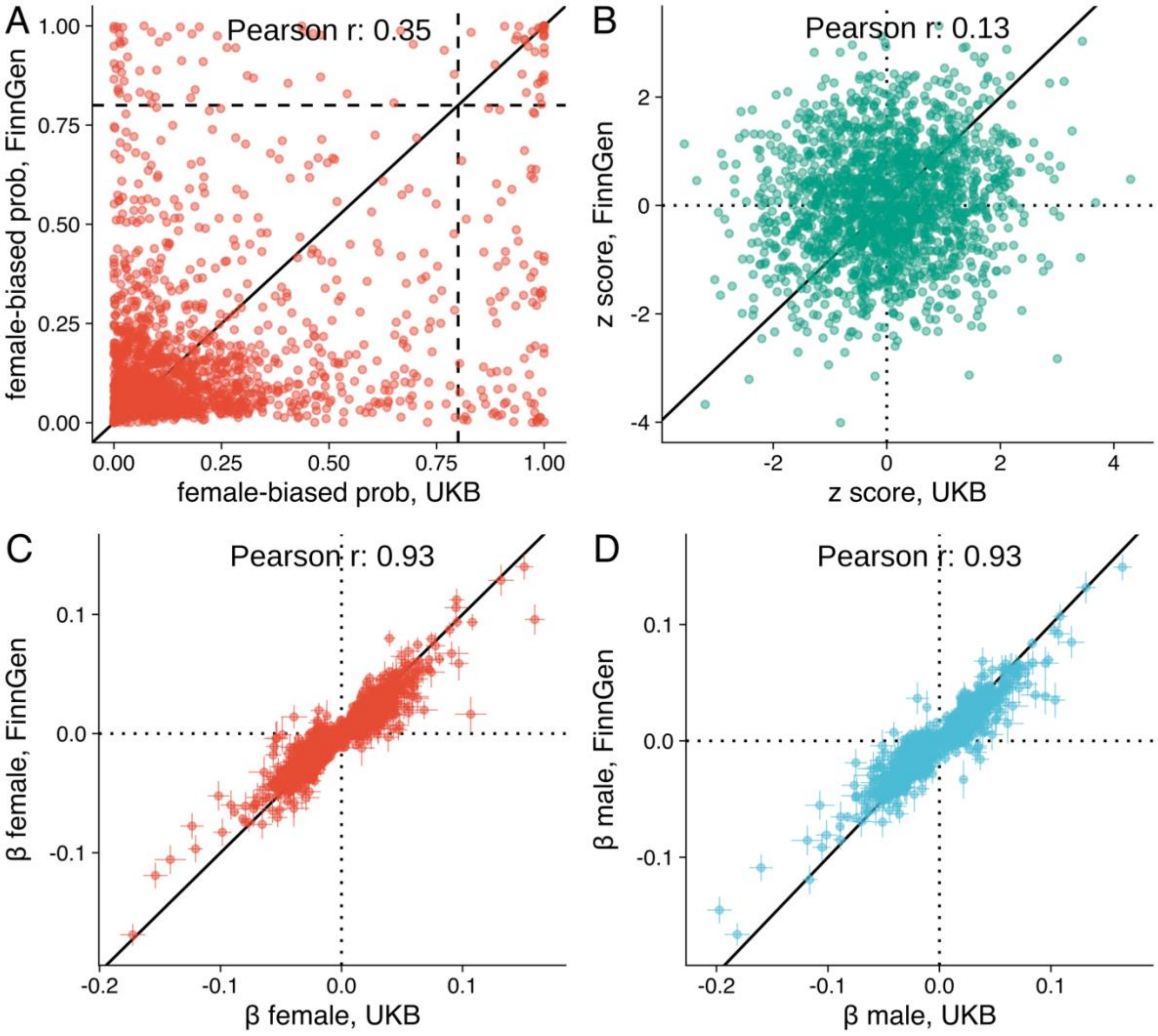

Figure S13. Comparison of UKB and FinnGen results.

The comparison in (A) female-biased probability, (B) sex difference z-score, (C) female effects (± SE) and (D) male effects (± SE) of lead variants identified in UKB height sex-combined GWAS in autosomes. Numerical values are reported in Tables S13 and S14.

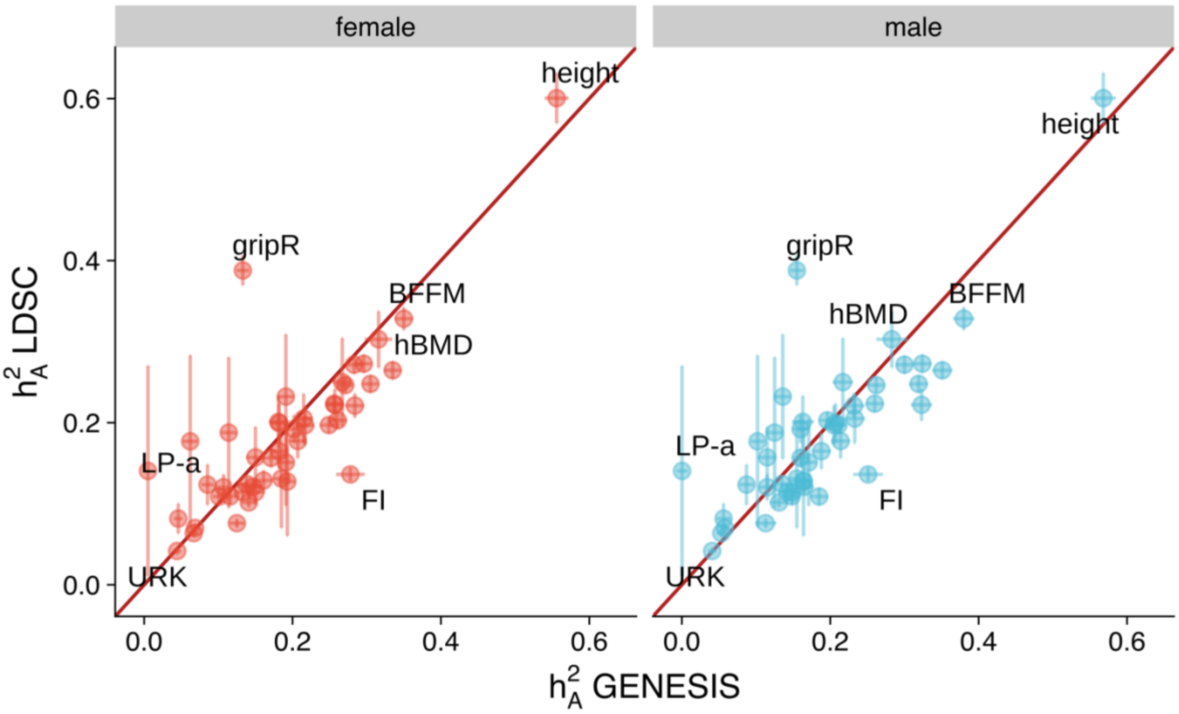

Figure S14. Comparison of estimates of autosomal heritability h_A^2 for 48 traits.

We compared the estimated h_A^2 by GENESIS to that estimated by LDSC using female and male summary statistics. Error bars indicate SE of h_A^2. Solid lines indicate equal estimates between the two methods. Numerical results are in Table S3. Abbreviations: hand grip strength, right (gripR), whole body fat-free mass (BFFM), heel bone mineral density T-score (hBMD), lipoprotein A (LP-a), fluid intelligence score (FI), potassium in urine (URK).

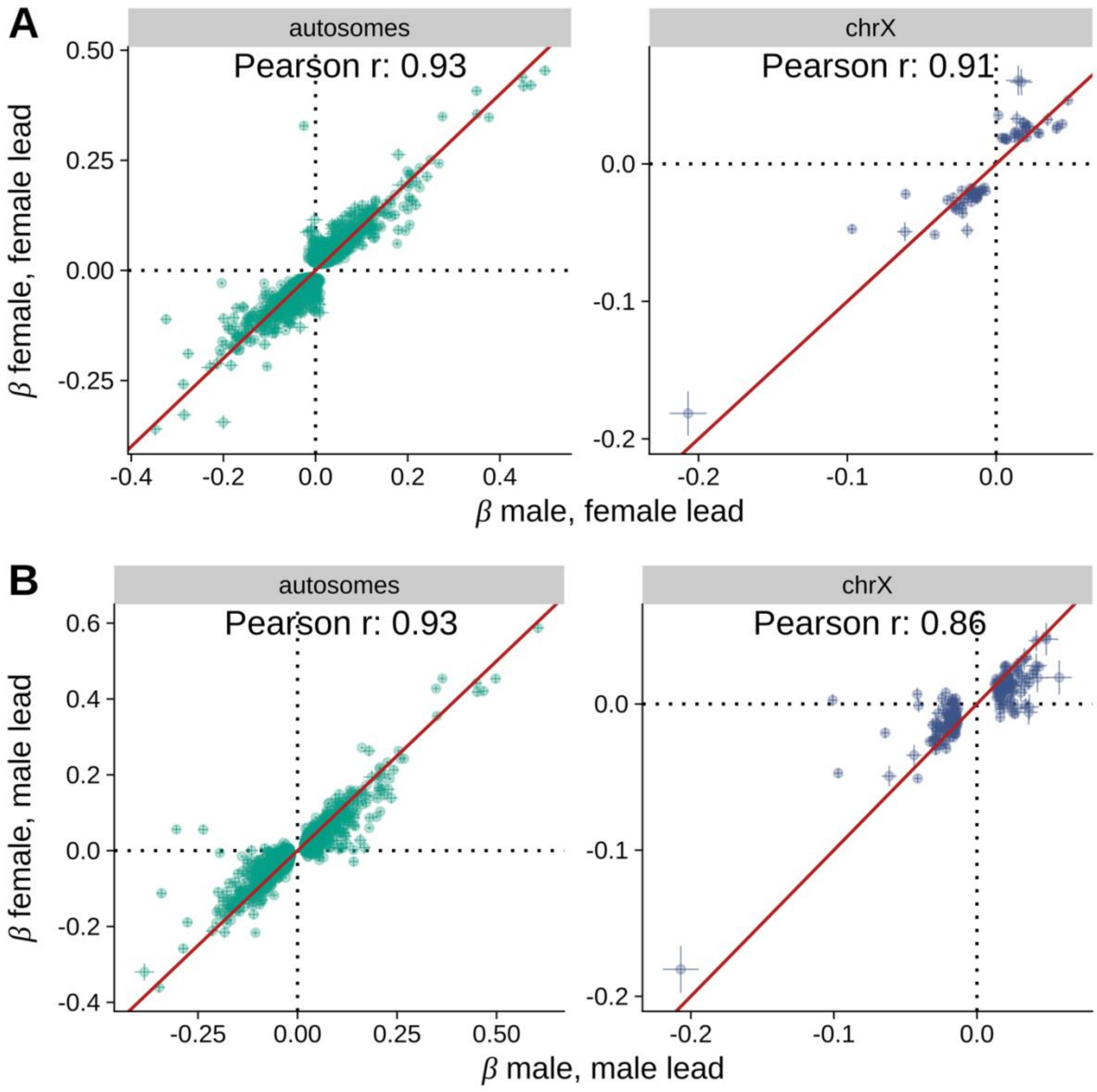

Figure S15. Comparison of male and female effects (± SE) of lead variants. The comparison of lead variants identified from (A) female-specific GWAS and (B) male-specific GWAS. The Pearson correlations after removing variants associated with testosterone, a trait known for its sex-specific effects and strong contribution from chrX, were 0.94 in autosomes and 0.92 in chrX in females and 0.96 in autosomes and 0.91 in chrX in males. The numerical values are reported in Table S7.

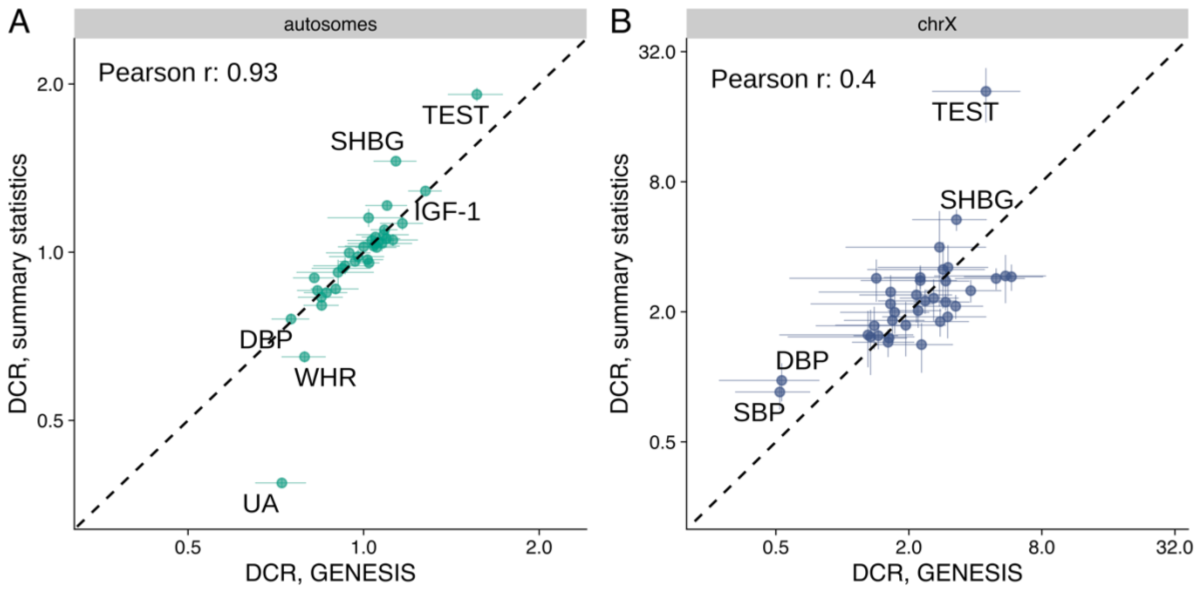

Figure S16. Comparison of autosomal and chrX dosage compensation ratio (DCR) using different methods.

We compared DCR (± SE) estimated using summary statistics and using h^2 estimated from GENESIS for (A) autosomes and (B) chrX. Numerical values are reported in Table S3. Abbreviations: testosterone (TEST), sex hormone-binding globulin (SHBG), insulin-like growth factor 1 (IGF-1), diastolic blood pressure (DBP), systolic blood pressure (SBP), waist-to-hip ratio (WHR), urate (UA).

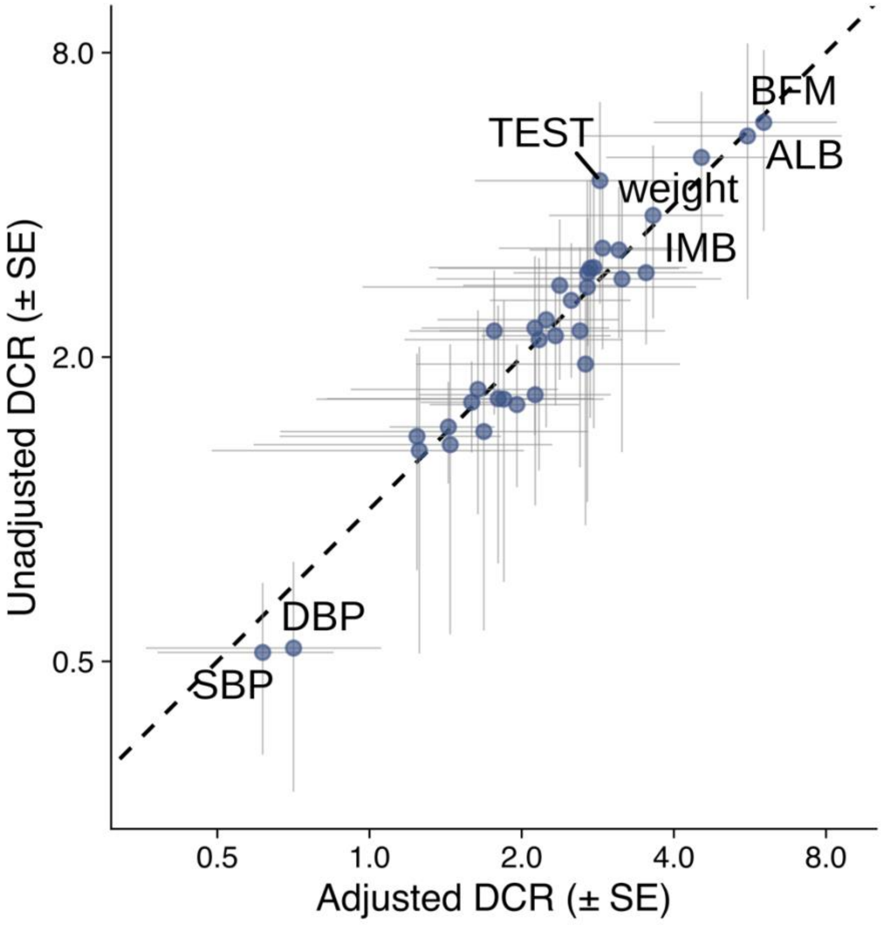

Figure S17. Comparison of chrX dosage compensation ratio (DCR) estimates with and without adjustment for autosomal DCR estimates.

Numerical values are reported in Table S3. Abbreviations: whole body fat mass (BFM), albumin (ALB), testosterone (TEST), impedance of whole body (IMB), diastolic blood pressure (DBP), systolic blood pressure (SBP).

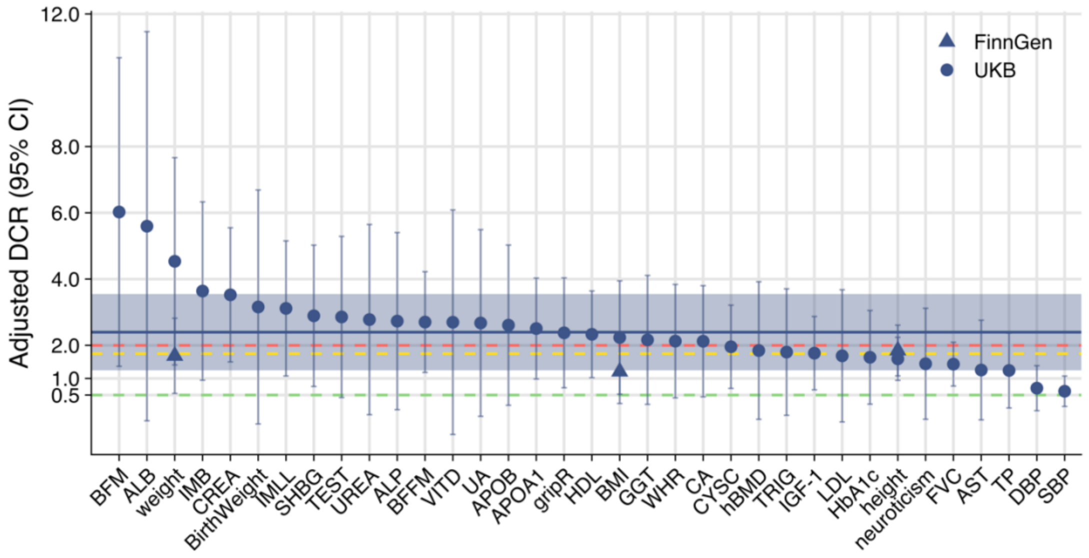

Figure S18. ChrX adjusted dosage compensation ratio (DCR) estimates with 95% CI (DCR ± 1.96*SE) of 35 traits with nonzero h_X^2 in both sexes.

The chrX DCR estimates were adjusted by autosomal DCR estimates. The solid blue line indicates the mean DCR and the shaded region indicates one standard deviation of the DCR point estimates of the traits. The red, yellow and green dashed lines indicate expectation under full XCI, 25% escape from XCI, and no XCI, respectively. Numerical values are reported in Table S3. Abbreviations: whole body fat mass (BFM), albumin (ALB), impedance of body (IMB), creatinine (CREA), impedance of leg, left (IMLL), sex hormone-binding globulin (SHBG), testosterone (TEST), urea (UREA), alkaline phosphatase (ALP), whole body fat-free mass (BFFM), vitamin D (VITD), urate (UA), apolipoprotein B (APOB), apolipoprotein (APOA1), hand grip strength, right (gripR), high-density lipoprotein cholesterol (HDL), body mass index (BMI), gamma glutamyl transferase (GGT), waist-to-hip ratio (WHR), calcium (CA), cystatin C (CYSC), heel bone mineral density T-score (hBMD), triglycerides (TRIG), insulin-like growth factor 1 (IGF-1), low-density lipoprotein cholesterol (LDL), glycated haemoglobin (HbA1c), forced vital capacity (FVC), aspartate

aminotransferase (AST), total protein (TP), diastolic blood pressure (DBP), systolic blood pressure (SBP).

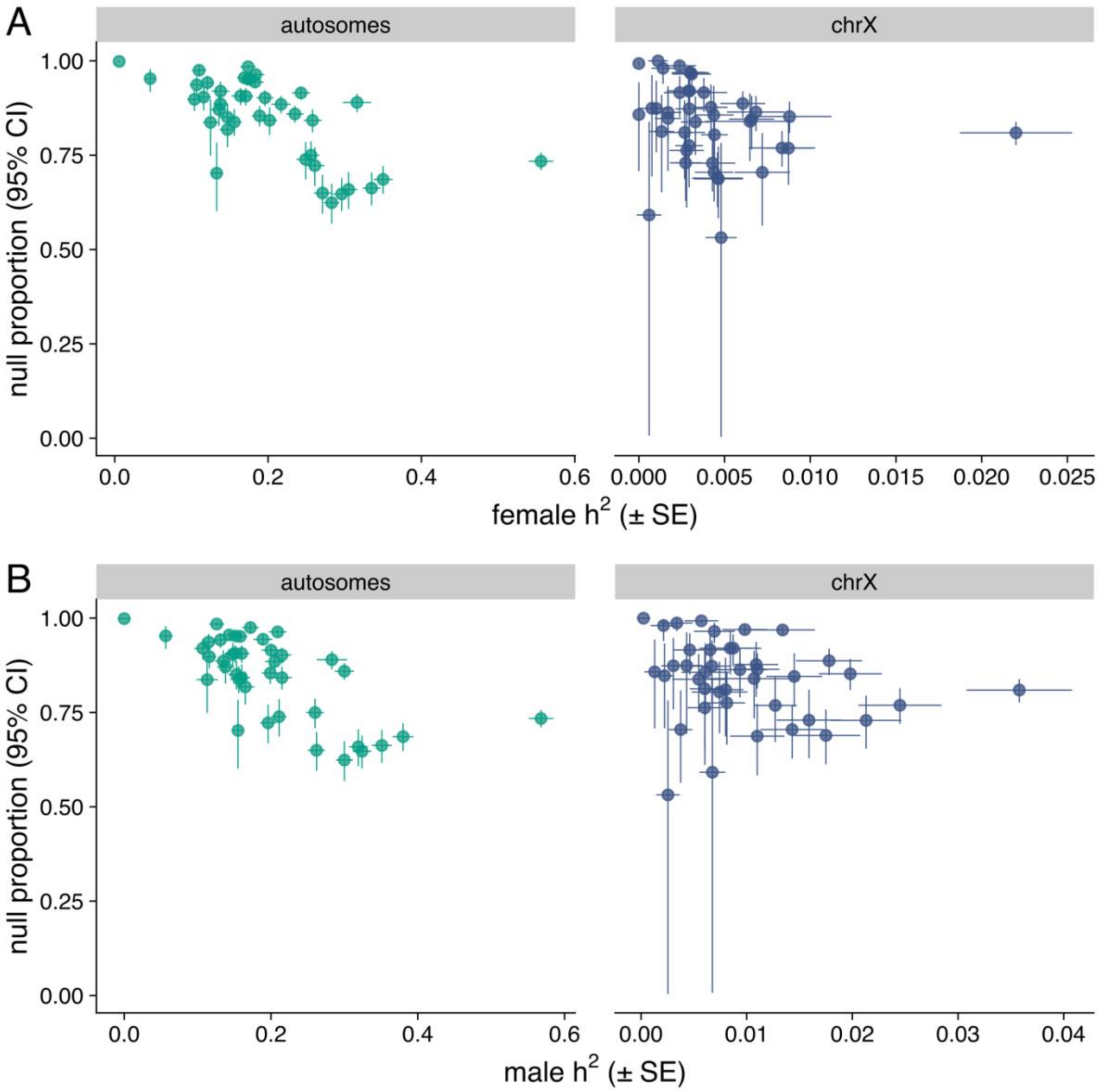

Figure S19. Comparison of estimated proportion of null variants using the mixture model of sex-specific effect sizes.

The comparisons were made with (A) female and (B) male h^2 estimated with GENESIS. Numerical values are reported in Tables S3 and S10.

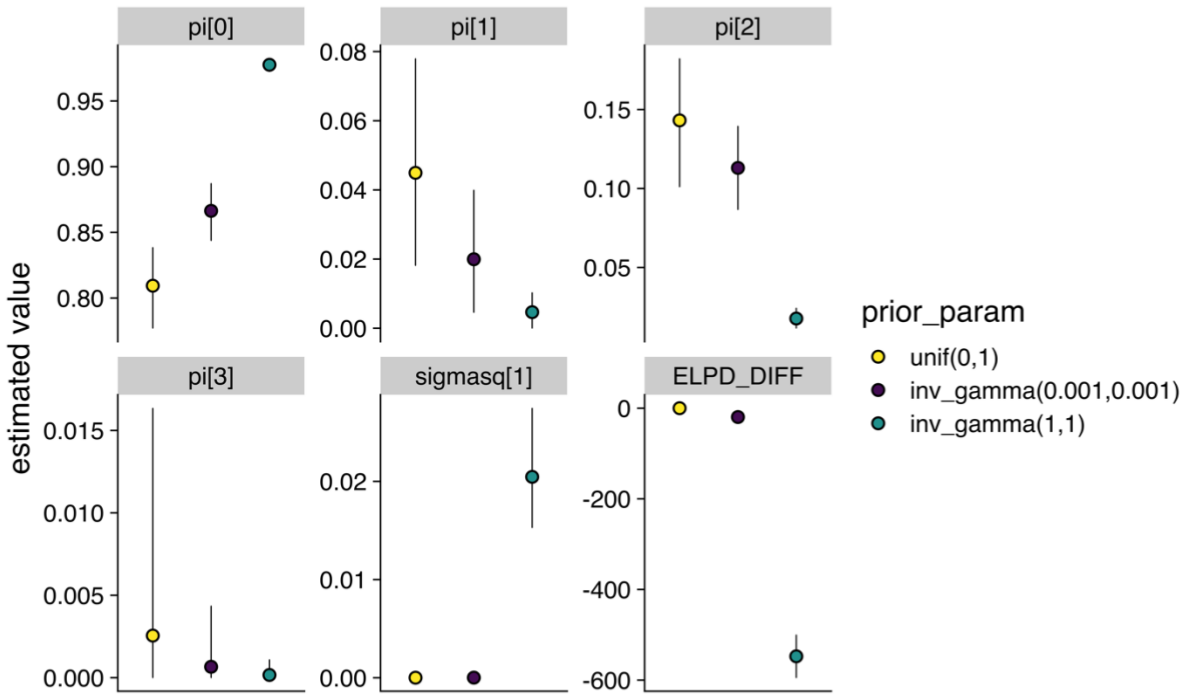

Figure S20. Comparison of different priors on σ^2 in four-component mixture model to classify variants between null, equal, female-biased and male-biased components.

Estimated posterior distributions of 5 parameters and ELPD-DIFF values with 3 different priors for σ^2. For estimated proportion parameters $\pi_1, \ldots, \pi_4$ and non-zero effects' variance σ^2 means and 2.5 to 97.5 percentile intervals are shown. For ELPD-DIFF, which is the difference in ELPD-LOO of each model relative to the model with the highest ELPD-LOO, is shown together with its SE. Here, unif(0,1) prior has the highest ELPD-LOO and is therefore preferred. Numeric values are reported in Tables S11 and S12. See https://mc-stan.org/loo/reference/loo_compare for a description of this model assessment approach.

Supplemental Tables

Table S1. Expected relationships of quantities between sexes and between chrX and autosomes under different genotype coding systems. Subscripts m and f denote males and females, respectively. Subscripts X and A denote chrX and autosomes, respectively. p, the minor allele frequency, here expected to be equal for effect alleles in chrX and in autosomes; G, genotype and $var(G)$ the genotype variance; β, the effect size of a variant when the trait Y is regressed on the genotype G; $var(\beta G)$, phenotypic variance explained by a variant; h^2, heritability defined as $var(\beta G)/var(Y)$, where $var(Y)$ is the total trait variance; a, the active allele effect.

<table>
<tr><td colspan="2">Genotype coding in chrX non-PAR</td><td colspan="2">Male: {0,2}
Female: {0,1,2}</td><td colspan="2">Male: {0,1}
Female: {0,1,2}</td><td colspan="2">Male: {0,1}
Female: {0,0.5,1}</td></tr>
<tr><td colspan="2">Genotype variance of chrX variants ($var(G)$)</td><td colspan="2">$var_{m,X} = 4p(1-p)$
$var_{f,X} = 2p(1-p)$</td><td colspan="2">$var_{m,X} = p(1-p)$
$var_{f,X} = 2p(1-p)$</td><td colspan="2">$var_{m,X} = p(1-p)$
$var_{f,X} = 0.5p(1-p)$</td></tr>
<tr><td colspan="2">Assuming full XCI and same active allele effect between sexes in the chrX ($a_f = a_m$)</td><td colspan="2">$\beta_{m,X}$, $\beta_{f,X}$ – half of active allele effect size
$\beta_{m,X} = \beta_{f,X} = \frac{a_X}{2}$
$var(\beta_{m,X} G_{m,X})$ $/var(\beta_{f,X} G_{f,X}) = 2$
$h^2_{m,X}/h^2_{f,X} = 2$</td><td colspan="2">$\beta_{m,X}$ – active allele effect size
$\beta_{f,X}$ – half of active allele effect size
$\beta_{m,X} = 2\beta_{f,X} = a_X$
$var(\beta_{m,X} G_{m,X})$ $/var(\beta_{f,X} G_{f,X}) = 2$
$h^2_{m,X}/h^2_{f,X} = 2$</td><td colspan="2">$\beta_{m,X}$, $\beta_{f,X}$ – active allele effect size
$\beta_{m,X} = \beta_{f,X} = a_X$
$var(\beta_{m,X} G_{m,X})$ $/var(\beta_{f,X} G_{f,X}) = 2$
$h^2_{m,X}/h^2_{f,X} = 2$</td></tr>
<tr><td colspan="2">Assumed effect relationship between chrX and autosomes</td><td>X = AA</td><td>X = A</td><td>X = AA</td><td>X = A</td><td>X = AA</td><td>X = A</td></tr>
<tr><td colspan="2">a_X/a_A</td><td>2</td><td>1</td><td>2</td><td>1</td><td>2</td><td>1</td></tr>
<tr><td rowspan="2">β_X/β_A</td><td>male</td><td>1</td><td>0.5</td><td>2</td><td>1</td><td>2</td><td>1</td></tr>
<tr><td>female</td><td>1</td><td>0.5</td><td>1</td><td>0.5</td><td>2</td><td>1</td></tr>
<tr><td rowspan="2">$var(\beta_X G_X)$ $/var(\beta_A G_A)$</td><td>male</td><td>2</td><td>0.5</td><td>2</td><td>0.5</td><td>2</td><td>0.5</td></tr>
<tr><td>female</td><td>1</td><td>0.25</td><td>1</td><td>0.25</td><td>1</td><td>0.25</td></tr>
<tr><td rowspan="2">h^2_X/h^2_A</td><td>male</td><td>2ϕ</td><td>$\phi/2$</td><td>2ϕ</td><td>$\phi/2$</td><td>2ϕ</td><td>$\phi/2$</td></tr>
<tr><td>female</td><td>ϕ</td><td>$\phi/4$</td><td>ϕ</td><td>$\phi/4$</td><td>ϕ</td><td>$\phi/4$</td></tr>
</table>

Table S2. Information on the UKB and FinnGen traits and the covariates used in GWAS.

Table S3. Estimated sex-specific and sex-combined h^2 in autosomes and chrX with GENESIS and LDSC. Sex-combined estimate is computed as average of sex-specific estimates. We tested if h^2 is different from zero (indicated by the “h2_x_pval” column) using GENESIS estimates and also report FDR adjusted *P*-values. "XI" is the chrX-to-autosome h^2 ratio, with "XI_se" the corresponding standard error (SE). We compared the male and female h^2 estimates in chrX and autosomes, with “pval_sex_diff_x” and “pval_sex_diff_a” reporting the corresponding *P*-values in chrX and autosomes, and “fdr_sex_diff_x” and “fdr_sex_diff_a” the corresponding FDR adjusted *P*-values. “DCR_GENESIS” is calculated based on GENESIS estimates for chrX and autosomes. “DCR_sumstats” is calculated using summary statistics.

Table S4. The effect size distribution estimates from GENESIS M2 model in autosomes and chrX with female summary statistics. The proportions of causal SNPs are in columns “pic.autosomes” and “pic.se.autosomes” for autosomes, and “pic.x” and “pic.se.x” for chrX. The per SNP h^2 are in columns “sigmasq.autosomes” and “sigmasq.se.autosomes” for autosomes, and “sigmasq.x” and “sigmasq.se.x” for chrX. The estimated $h^2$are in “h2.autosomes” and “h2.se.autosomes” for autosomes and “h2.x” and “h2.se.x” for chrX, which are the same as the estimated female h^2 by GENESIS in Table S3. The estimated number of causal SNPs are in columns “nbr.sSNP.autosomes” and “nbr.sSNP.se.autosomes” for autosomes and “nbr.sSNP.x” and “nbr.sSNP.se.x” for chrX.

Table S5. The effect size distribution estimates from GENESIS M2 model in autosomes and chrX with male summary statistics. The proportions of causal SNPs are in columns “pic.autosomes” and “pic.se.autosomes” for autosomes, and

“pic.x” and “pic.se.x” for chrX. The per SNP h^2 are in columns “sigmasq.autosomes” and “sigmasq.se.autosomes” for autosomes, and “sigmasq.x” and “sigmasq.se.x” for chrX. The estimated $h^2$are in “h2.autosomes” and “h2.se.autosomes” for autosomes and “h2.x” and “h2.se.x” for chrX, which are the same as the estimated male h^2 by GENESIS in Table S3. The estimated number of causal SNPs are in columns “nbr.sSNP.autosomes” and “nbr.sSNP.se.autosomes” for autosomes and “nbr.sSNP.x” and “nbr.sSNP.se.x” for chrX.

Table S6. The number of sex-specific LD-independent lead variants (*P*-value < 5×10^{-8}) in autosomes and chrX.

trait	chrX		autosomes	
	male	female	male	female
ALB	5	1	85	101
ALP	3	3	167	189
ALT	1	0	95	97
APOA1	5	0	113	144
APOB	3	2	118	162
AST	2	0	105	135
BFFM	20	5	293	307
BFM	9	1	139	163
BirthWeight	2	1	21	48
BMI	8	1	150	177
CA	2	0	90	109
CHOL	3	2	130	172
CREA	12	6	158	232
CRP	0	0	55	90
CYSC	11	4	140	185
DBIL	1	0	72	38
DBP	0	1	93	186
FI	0	0	5	12
FVC	3	3	142	169
GGT	5	1	156	179
GLU	0	0	44	54
gripR	3	0	53	50
HbA1c	5	0	180	206
hBMD	5	2	130	194
HDL	6	1	123	156
height	25	13	463	521
IGF-1	15	6	224	215
IMB	11	2	210	245
IMLL	8	1	188	227
LDL	3	2	120	156
LP-a	0	0	2	4
neuroticism	0	0	11	23
PHOS	2	1	75	74
pulseAuto	2	0	84	117

SBP	1	0	96	188
SHBG	3	1	179	150
TBIL	2	0	70	66
TEST	8	2	146	80
TP	2	0	78	104
TRIG	3	1	82	137
UA	3	0	81	132
UCR	0	0	3	8
UREA	6	2	84	83
URK	0	0	0	3
URNA	1	0	3	8
VITD	1	0	27	27
weight	11	2	193	213
WHR	2	2	67	201

Table S7. Summary statistics of lead variants identified in sex-specific GWAS for all traits. In the allelic effect columns, "ALLELE_EFF_FEMALE" and "ALLELE_EFF_MALE" and corresponding SE in columns "ALLELE_SE_FEMALE" and "ALLELE_SE_MALE" were the GWAS effect estimates "BETA_FEMALE" and "BETA_MALE", "SE_FEMALE" and "SE_MALE" multiplied by two for variants in the non-PAR region to correspond the active allele effects described in the main text. The samples included in each GWAS were indicated in the column "SAMPLE", which is "female" for female-specific GWAS, "male" for full sample sized male-specific GWAS, "downsize male" for downsized male-specific GWAS in the non-PAR region. "ALLELE1" was used as the effect allele and "ALLELE0" the reference allele in GWAS. "A1FREQ_FEMALE" and "A1FREA_MALE" are the frequencies of the effective allele in females and males, respectively. And "MAF_FEMALE" AND "MAF_MALE" are the minor allele frequencies in females and males, respectively. "P_BOLT_LMM_INF_FEMALE" and "P_BOLT_LMM_INF_MALE" are the BOLT-LMM infinitesimal mixed model association test *P*-values in females and males. "Consequence" is the VEP predicted consequence of the SNP and corresponding severity of the consequence "ConsScore" was reported by CADD, and the affected gene were given with Stable ID ("GeneID") and gene name ("GeneName"). The CADD prediction of the pathogenicity of each variant is in columns "RawScore" for unscaled C-score and "PHRED" for scaled C-score.

Table S8. Estimated number of LD blocks and average length of the LD blocks per chromosome and aggregated over all autosomes.

Chromosome	Chromosome length (bp)	Number of LD blocks	Average length of LD blocks (bp)
1	249250621	200	1246150.0
2	243199373	220	1105355.4
3	198022430	175	1130869.6
4	191154276	179	1067227.1
5	180915260	151	1197965.9
6	171115067	168	1017794.2
7	159138663	138	1152996.4
8	146364022	138	1060087.3
9	141213431	115	1227271.4
10	135534747	125	1083710.9
11	135006516	130	1037379.2
12	133851895	123	1087654.5
13	115169878	91	1055931.4
14	107349540	80	1103617.2
15	102531392	68	1213540.8
16	90354753	71	1270882.0
17	81195210	65	1249114.0
18	78077248	68	1147152.3
19	59128983	63	937366.8
20	63025520	61	1031194.0
21	48129895	32	1209637.2
22	51304566	34	1035118.4
Autosomes	2985940020	2495	1120667.0
chrX, non-PAR	152301523	71	2144094.0
chrX, PAR	2969037	5	593267.0
chrX	155270560	76	2042066.0

Table S9. Posterior probabilities of XCI scenarios for each trait based on sex-specific h^2 in chrX and in autosomes.

trait	data	pp.F.XCI	pp.E.XCI	pp.N.XCI	group	chr.type
ALB	UKB	0.7420	0.2324	0.0256	uncategorized	chrX
ALP	UKB	0.7683	0.2301	0.0016	uncategorized	chrX
APOA1	UKB	0.7599	0.2398	0.0003	uncategorized	chrX
APOB	UKB	0.7383	0.2345	0.0272	uncategorized	chrX
AST	UKB	0.4995	0.1463	0.3542	uncategorized	chrX
BFFM	UKB	0.5061	0.4939	0.0000	uncategorized	chrX
BFM	UKB	0.7509	0.2481	0.0010	uncategorized	chrX
BirthWeight	UKB	0.7058	0.2262	0.0680	uncategorized	chrX
BMI	UKB	0.7110	0.2838	0.0052	uncategorized	chrX
CA	UKB	0.7630	0.2191	0.0179	uncategorized	chrX
CREA	UKB	0.6944	0.3055	0.0001	uncategorized	chrX
CYSC	UKB	0.5932	0.4040	0.0028	uncategorized	chrX
DBP	UKB	0.0051	0.0145	0.9804	N-XCI	chrX
FVC	UKB	0.4084	0.5910	0.0006	uncategorized	chrX
GGT	UKB	0.7505	0.2254	0.0241	uncategorized	chrX
gripR	UKB	0.7824	0.2161	0.0014	uncategorized	chrX
HbA1c	UKB	0.7062	0.2680	0.0259	uncategorized	chrX
hBMD	UKB	0.5318	0.3162	0.1521	uncategorized	chrX
HDL	UKB	0.6468	0.3530	0.0002	uncategorized	chrX
height	UKB	0.0051	0.9949	0.0000	E-XCI	chrX
IGF-1	UKB	0.4504	0.5496	0.0000	uncategorized	chrX
IMB	UKB	0.7262	0.2736	0.0002	uncategorized	chrX
IMLL	UKB	0.7486	0.2503	0.0010	uncategorized	chrX
LDL	UKB	0.5297	0.1718	0.2986	uncategorized	chrX
neuroticism	UKB	0.5108	0.1559	0.3333	uncategorized	chrX
SBP	UKB	0.0079	0.0101	0.9820	N-XCI	chrX
SHBG	UKB	0.7745	0.2240	0.0015	uncategorized	chrX
TEST	UKB	0.7622	0.2337	0.0041	uncategorized	chrX
TP	UKB	0.5438	0.2488	0.2073	uncategorized	chrX
TRIG	UKB	0.5818	0.2052	0.2130	uncategorized	chrX
UA	UKB	0.6913	0.2040	0.1048	uncategorized	chrX
UREA	UKB	0.7470	0.2349	0.0182	uncategorized	chrX
VITD	UKB	0.6773	0.1936	0.1291	uncategorized	chrX
weight	UKB	0.7430	0.2569	0.0000	uncategorized	chrX
WHR	UKB	0.6289	0.2651	0.1060	uncategorized	chrX
height	FinnGen	0.0036	0.9964	0.0000	E-XCI	chrX
BMI	FinnGen	0.5496	0.3535	0.0969	uncategorized	chrX
weight	FinnGen	0.6006	0.3952	0.0042	uncategorized	chrX
ALB	UKB	0.5174	0.4826	0.0000	uncategorized	autosomes
ALP	UKB	0.9076	0.0924	0.0000	F-XCI	autosomes
APOA1	UKB	0.7285	0.2715	0.0000	uncategorized	autosomes
APOB	UKB	0.2516	0.7484	0.0000	uncategorized	autosomes

AST	UKB	0.6604	0.3396	0.0000	uncategorized	autosomes
BFFM	UKB	0.9980	0.0020	0.0000	F-XCI	autosomes
BFM	UKB	0.7078	0.2922	0.0000	uncategorized	autosomes
BirthWeight	UKB	0.3957	0.6043	0.0000	uncategorized	autosomes
BMI	UKB	0.9886	0.0114	0.0000	F-XCI	autosomes
CA	UKB	0.6183	0.3817	0.0000	uncategorized	autosomes
CREA	UKB	0.1309	0.8691	0.0000	E-XCI	autosomes
CYSC	UKB	0.1272	0.8728	0.0000	E-XCI	autosomes
DBP	UKB	0.0336	0.9664	0.0000	E-XCI	autosomes
FVC	UKB	0.8812	0.1188	0.0000	F-XCI	autosomes
GGT	UKB	0.6035	0.3965	0.0000	uncategorized	autosomes
gripR	UKB	0.9254	0.0746	0.0000	F-XCI	autosomes
HbA1c	UKB	0.8308	0.1692	0.0000	F-XCI	autosomes
hBMD	UKB	0.3931	0.6069	0.0000	uncategorized	autosomes
HDL	UKB	0.5050	0.4950	0.0000	uncategorized	autosomes
height	UKB	0.9992	0.0008	0.0000	F-XCI	autosomes
IGF-1	UKB	0.9997	0.0003	0.0000	F-XCI	autosomes
IMB	UKB	0.9892	0.0108	0.0000	F-XCI	autosomes
IMLL	UKB	0.9809	0.0191	0.0000	F-XCI	autosomes
LDL	UKB	0.2259	0.7741	0.0000	uncategorized	autosomes
neuroticism	UKB	0.4229	0.5771	0.0000	uncategorized	autosomes
SBP	UKB	0.1393	0.8607	0.0000	E-XCI	autosomes
SHBG	UKB	0.9378	0.0622	0.0000	F-XCI	autosomes
TEST	UKB	0.9942	0.0058	0.0000	F-XCI	autosomes
TP	UKB	0.8147	0.1853	0.0000	F-XCI	autosomes
TRIG	UKB	0.4161	0.5839	0.0000	uncategorized	autosomes
UA	UKB	0.1048	0.8952	0.0000	E-XCI	autosomes
UREA	UKB	0.7124	0.2876	0.0000	uncategorized	autosomes
VITD	UKB	0.4913	0.5087	0.0000	uncategorized	autosomes
weight	UKB	0.9959	0.0041	0.0000	F-XCI	autosomes
WHR	UKB	0.1160	0.8840	0.0000	E-XCI	autosomes
height	FinnGen	0.7577	0.2423	0.0000	uncategorized	autosomes
BMI	FinnGen	0.7305	0.2695	0.0000	uncategorized	autosomes
weight	FinnGen	0.8518	0.1482	0.0000	F-XCI	autosomes

Table S10. Estimated parameters, proportions of null (p[0]), female-biased (p[1]), equal (p[2]) and male-biased (p3) components and σ^2, of all traits in four-component sex bias mixture model. We report the summaries of the parameters: mean (“mean”), Monte Carlo standard errors (“se_mean”), standard deviations (“sd”), quantiles (“2.5%”,”50%” and “97.5%”), effective sample sizes (“n_eff”), split Rhats (“Rhat”). The “Data” column indicates whether UKB or FinnGen summary statistics were used.

Table S11. The estimates (mean), and 2.5, 50, 97.5 percentile ("2.5%","50%" and "97.5%") of each parameter with different priors for sigmasq ("prior_param"). The analyses were performed using UKB height summary statistics in chrX, as well effective sample sizes ("n_eff") and split Rhats ("Rhat") from the fit that used to assess the convergence.

prior_param	parameter	mean	2.5%	50%	97.5%	N_eff	Rhat
unif (0,1)	p[0]	0.8094	0.7771	0.8097	0.8385	3210.04	1.0010
unif (0,1)	p[1]	0.0449	0.0181	0.0437	0.0779	3533.35	0.9999
unif (0,1)	p[2]	0.1431	0.1010	0.1436	0.1822	3854.99	1.0000
unif (0,1)	p[3]	0.0026	0.0000	0.0005	0.0163	5113.12	1.0004
unif(0,1)	sigmasq[1]	9.01E-6	7.53E-6	8.96E-6	1.08E-5	3064.71	1.0003
inv_gamma (0.001,0.001)	p[0]	0.8664	0.8437	0.8668	0.8872	3435.81	1.0005
inv_gamma (0.001,0.001)	p[1]	0.0199	0.0046	0.0191	0.0399	4327.91	1.0004
inv_gamma (0.001,0.001)	p[2]	0.1130	0.0866	0.1130	0.1395	3984.83	1.0006
inv_gamma (0.001,0.001)	p[3]	0.0007	0.0000	0.0001	0.0043	5034.89	1.0009
inv_gamma (0.001,0.001)	sigmasq[1]	1.66E-5	1.40E-5	1.65E-5	1.97E-5	3254.32	1.0010
inv_gamma (1,1)	p[0]	0.9775	0.9725	0.9776	0.9822	3959.17	1.0010
inv_gamma (1,1)	p[1]	0.0046	0.0000	0.0045	0.0103	1959.27	1.0015
inv_gamma (1,1)	p[2]	0.0177	0.0115	0.0175	0.0243	2679.20	1.0016
inv_gamma (1,1)	p[3]	0.0002	0.0000	0.0000	0.0011	6352.75	1.0000
inv_gamma (1,1)	sigmasq[1]	2.05E-2	1.53E-2	2.02E-2	2.75E-2	4273.61	1.0002

Table S12. Leave-one-out cross validation of the three prior distributions using the "loo" package. The expected log posterior density ("elpd_loo") and corresponding standard error (SE), and the difference between each prior and the best prior ("elpd_diff") and the SE of the difference. The values of "p_loo" and "looic" and corresponding SE are also included for reference.

prior_param	elpd_diff (SE)	elpd_loo (SE)	p_loo (SE)	looic (SE)
unif (0,1)	0 (0)	41594.1 (85.9)	5.2 (1.6)	-83188.2 (171.8)
inv_gamma (0.001,0.001)	-19.5 (10.1)	41574.6 (85.3)	3.4 (0.8)	-83149.2 (170.7)
inv_gamma (1,1)	-547.5 (47.2)	41046.5 (118.2)	3.1 (0.5)	-82093.1 (236.4)

Table S13. Summary statistics of lead variants identified in the conditional analyses from sex-combined GWAS and their posterior probabilities within each sex-biased component and their component assignments, as well as sex difference z-score ("SEX_DIFF_Z") of each variant. "ALLELE1" was used as the effect allele and "ALLELE0" the reference allele in GWAS. The "INFO" contains imputation quality score of variants. Suffixes are used to indicate if the estimation was performed in the sex-combined ("_COMB"), female ("_FEMALE") or male ("_MALE") population. "A1FREQ" contains the allele frequency of the effect allele in the sample and "MAF" the minor allele frequency in the sample. "BETA", "SE", "CHISQ_BOLT_LMM_INF", "P_BOLT_LMM_INF" are the estimated effect size, corresponding SE, chi-square statistics, and *P*-values from BOLT-LMM infinitesimal mixed model, respectively. We report the posterior probabilities of each lead SNP in the null effect ("p[0]"), the female-biased effect("p[1]"), equal effect ("p[2]") and male-biased effect ("p[3]") and the assigned component ("COMPONENT").

Table S14. Summary statistics estimated in FinnGen release 10 of height-associated lead variants identified in UKB sex-combined GWAS and their posterior probabilities within each sex-biased component and their component assignments, as well as sex difference z-score ("SEX_DIFF_Z") of each variant. "ALLELE1" was used as the effect allele and "ALLELE0" the reference allele in GWAS. Suffixes are used to indicate if the estimation were performed in female ("_FEMALE") or male ("_MALE") population. "A1FREQ" contains allele frequency of the effect allele in the sample. "BETA", "SE", "P" are the estimated effect size, corresponding SE and *P*-values, respectively. We report the posterior probabilities of each lead SNP in the null effect ("p[0]"), the female-biased effect("p[1]"), equal effect ("p[2]") and male-biased effect ("p[3]") and the assigned component ("COMPONENT").

Supplemental Acknowledgement

The FinnGen project is funded by two grants from Business Finland (HUS 4685/31/2016 and UH 4386/31/2016) and the following industry partners: AbbVie Inc., AstraZeneca UK Ltd, Biogen MA Inc., Bristol Myers Squibb (and Celgene Corporation & Celgene International II Sàrl), Genentech Inc., Merck Sharp & Dohme LCC, Pfizer Inc., GlaxoSmithKline Intellectual Property Development Ltd., Sanofi US Services Inc., Maze Therapeutics Inc., Janssen Biotech Inc, Novartis AG, and Boehringer Ingelheim International GmbH.

Following biobanks are acknowledged for delivering biobank samples to FinnGen: Auria Biobank (www.auria.fi/biopankki), THL Biobank (www.thl.fi/biobank), Helsinki Biobank (www.helsinginbiopankki.fi), Biobank Borealis of Northern Finland (https://www.ppshp.fi/Tutkimus-ja-opetus/Biopankki/Pages/Biobank-Borealis-briefly-in-English.aspx), Finnish Clinical Biobank Tampere (www.tays.fi/en-US/Research_and_development/Finnish_Clinical_Biobank_Tampere), Biobank of Eastern Finland (www.ita-suomenbiopankki.fi/en), Central Finland Biobank (www.ksshp.fi/fi-FI/Potilaalle/Biopankki), Finnish Red Cross Blood Service Biobank (www.veripalvelu.fi/verenluovutus/biopankkitoiminta), Terveystalo Biobank (www.terveystalo.com/fi/Yritystietoa/Terveystalo-Biopankki/Biopankki/) and Arctic Biobank (https://www.oulu.fi/en/university/faculties-and-units/faculty-medicine/northern-finland-birth-cohorts-and-arctic-biobank). All Finnish Biobanks are members of BBMRI.fi infrastructure (https://www.bbmri-eric.eu/national-nodes/finland/). Finnish Biobank Cooperative -FINBB (https://finbb.fi/) is the coordinator of BBMRI-ERIC operations in Finland. The Finnish biobank data can be accessed through the Fingenious® services (https://site.fingenious.fi/en/) managed by FINBB.

Supplemental References

1. Purcell, S., Neale, B., Todd-Brown, K., Thomas, L., Ferreira, M.A.R., Bender, D., Maller, J., Sklar, P., de Bakker, P.I.W., Daly, M.J., et al. (2007). PLINK: A Tool Set for Whole-Genome Association and Population-Based Linkage Analyses. Am. J. Hum. Genet. *81*, 559–575. https://doi.org/10.1086/519795.

2. Sidorenko, J., Kassam, I., Kemper, K.E., Zeng, J., Lloyd-Jones, L.R., Montgomery, G.W., Gibson, G., Metspalu, A., Esko, T., Yang, J., et al. (2019). The effect of X-linked dosage compensation on complex trait variation. Nat. Commun. *10*, 3009. https://doi.org/10.1038/s41467-019-10598-y.

3. Gao, F., Chang, D., Biddanda, A., Ma, L., Guo, Y., Zhou, Z., and Keinan, A. (2015). XWAS: A Software Toolset for Genetic Data Analysis and Association Studies of the X Chromosome. J. Hered. *106*, 666–671. https://doi.org/10.1093/jhered/esv059.

4. Keur, N., Ricaño-Ponce, I., Kumar, V., and Matzaraki, V. (2022). A systematic review of analytical methods used in genetic association analysis of the X-chromosome. Brief. Bioinform. *23*, bbac287. https://doi.org/10.1093/bib/bbac287.

5. Tukiainen, T., Villani, A.-C., Yen, A., Rivas, M.A., Marshall, J.L., Satija, R., Aguirre, M., Gauthier, L., Fleharty, M., Kirby, A., et al. (2017). Landscape of X chromosome inactivation across human tissues. Nature *550*, 244–248. https://doi.org/10.1038/nature24265.

6. Marchini, J., and Howie, B. (2010). Genotype imputation for genome-wide association studies. Nat. Rev. Genet. *11*, 499–511. https://doi.org/10.1038/nrg2796.

7. Bulik-Sullivan, B.K., Loh, P.-R., Finucane, H.K., Ripke, S., Yang, J., Patterson, N., Daly, M.J., Price, A.L., and Neale, B.M. (2015). LD Score regression distinguishes confounding from polygenicity in genome-wide association studies. Nat. Genet. *47*, 291–295. https://doi.org/10.1038/ng.3211.

8. McLaren, W., Gil, L., Hunt, S.E., Riat, H.S., Ritchie, G.R.S., Thormann, A., Flicek, P., and Cunningham, F. (2016). The Ensembl Variant Effect Predictor. Genome Biol. *17*, 122. https://doi.org/10.1186/s13059-016-0974-4.

9. Rentzsch, P., Witten, D., Cooper, G.M., Shendure, J., and Kircher, M. (2019). CADD: predicting the deleteriousness of variants throughout the human genome. Nucleic Acids Res. *47*, D886–D894. https://doi.org/10.1093/nar/gky1016.

10. Gorlov, I.P., and Amos, C.I. (2023). Why does the X chromosome lag behind autosomes in GWAS findings? PLOS Genet. *19*, e1010472. https://doi.org/10.1371/journal.pgen.1010472.

11. Gottipati, S., Arbiza, L., Siepel, A., Clark, A.G., and Keinan, A. (2011). Analyses of X-linked and autosomal genetic variation in population-scale whole genome sequencing. Nat. Genet. *43*, 741–743. https://doi.org/10.1038/ng.877.

12. Flynn, E., Tanigawa, Y., Rodriguez, F., Altman, R.B., Sinnott-Armstrong, N., and Rivas, M.A. (2021). Sex-specific genetic effects across biomarkers. Eur. J. Hum. Genet. *29*, 154–163. https://doi.org/10.1038/s41431-020-00712-w.

13. Zhu, C., Ming, M.J., Cole, J.M., Edge, M.D., Kirkpatrick, M., and Harpak, A. (2023). Amplification is the primary mode of gene-by-sex interaction in complex human traits. Cell Genomics *3*, 100297. https://doi.org/10.1016/j.xgen.2023.100297.

14. Leinonen, J.T., Mars, N., Lehtonen, L.E., Ahola-Olli, A., Ruotsalainen, S., Lehtimäki, T., Kähönen, M., Raitakari, O., Piltonen, T., Daly, M., et al. (2023). Genetic analyses implicate complex links between adult testosterone levels and health and disease. Commun. Med. *3*, 1–15. https://doi.org/10.1038/s43856-022-00226-0.

15. Patten, M.M. (2019). The X chromosome favors males under sexually antagonistic selection. Evolution *73*, 84–91. https://doi.org/10.1111/evo.13646.

16. He, H., Gu, X., Xu, W., Yang, D., Wang, X., and Su, Y. (2013). Krüppel-like factor 8 is a novel androgen receptor co-activator in human prostate cancer. Acta Pharmacol. Sin. *34*, 282–288. https://doi.org/10.1038/aps.2012.130.

17. Zhuang, X., Feng, X., Tang, W., Zhu, J., Li, M., Li, J., Zheng, X., Li, R., Liu, P., and Qiao, J. (2021). FAM9B serves as a novel meiosis-related protein localized in meiotic chromosome cores and is associated with human gametogenesis. PLOS ONE *16*, e0257248. https://doi.org/10.1371/journal.pone.0257248.

18. Pirastu, N., Joshi, P.K., de Vries, P.S., Cornelis, M.C., McKeigue, P.M., Keum, N., Franceschini, N., Colombo, M., Giovannucci, E.L., Spiliopoulou, A., et al. (2017). GWAS for male-pattern baldness identifies 71 susceptibility loci explaining 38% of the risk. Nat. Commun. *8*, 1584. https://doi.org/10.1038/s41467-017-01490-8.

19. Henne, S.K., Aldisi, R., Sivalingam, S., Hochfeld, L.M., Borisov, O., Krawitz, P.M., Maj, C., Nöthen, M.M., and Heilmann-Heimbach, S. (2023). Analysis of 72,469 UK Biobank exomes links rare variants to male-pattern hair loss. Nat. Commun. *14*, 5492. https://doi.org/10.1038/s41467-023-41186-w.

20. Natarajan, P., Pampana, A., Graham, S.E., Ruotsalainen, S.E., Perry, J.A., de Vries, P.S., Broome, J.G., Pirruccello, J.P., Honigberg, M.C., Aragam, K., et al. (2021). Chromosome Xq23 is associated with lower atherogenic lipid concentrations and favorable cardiometabolic indices. Nat. Commun. *12*, 2182. https://doi.org/10.1038/s41467-021-22339-1.
